# NDR2 regulates non-small cell lung cancer cell migration under starvation by supporting autophagosome biogenesis through LC3 and ATG9A regulation

**DOI:** 10.1038/s41420-025-02889-9

**Published:** 2025-12-13

**Authors:** Tiphaine Biojout, Emmanuel Bergot, Jasmine Taylor, Dimitri Leite Ferreira, Nathalie Colloc’h, Marc Riffet, Nicolas Elie, Maelle Guyot, Céline Bazille, Jérôme Levallet, Guénaëlle Levallet

**Affiliations:** 1 Université de Caen Normandie, CNRS, Normandie Université, ISTCT UMR6030, GIP CYCERON, Caen, France; 2 Centre Hospitalier Universitaire de Caen, Service de Pneumologie et Oncologie Thoracique, Caen, France; 3 Centre Hospitalier Universitaire de Caen, Service d’Anatomie et Cytologie pathologiques, Caen, France; 4 Université de Caen Normandie, Service Unit EMERODE, Centre de Microscopie Appliquée à la Biologie, CMABio³, Caen, France; 5 Université de Caen Normandie, Federative Structure 4207 “Normandie Oncologie”, Service Unit PLATON, Virtual’His platform, Caen, France

**Keywords:** Lung cancer, Non-small-cell lung cancer, Macroautophagy

## Abstract

Non-small cell lung cancer (NSCLC) is characterized by the deregulation of the Hippo kinase NDR2 and high basal autophagic activity. NDR2 promotes autophagy-driven tumor growth in some cancers, but evidence in lung cancer is lacking. Human bronchial epithelial tumor cell (HBEC) lines H2030, H2030-BrM3, and H1299, with or without NDR2 depletion via siRNA or shRNA, were cultured for up to 24 h in the presence or absence of serum, and with or without the autophagosome–lysosome fusion inhibitor chloroquine (CQ). Autophagosome biogenesis, migration and Golgi apparatus functionality were analyzed. Serum deprivation of HBECs silences the expression of NDR1 but not NDR2. As shown by the increased expression of the autophagosome marker LC3-II, NDR2 participates to the formation and distribution of phagophores/autophagosomes in HBECs in an ATG9A-dependent manner. NDR2 is required for cargos degradation since its depletion disrupts lysosomal trafficking and/or fusion with autophagosomes. Finally, NDR2 silencing inhibits filopodia formation and cell polarization during HBEC migration under serum deprivation by disrupting Golgi repositioning to the leading edge, a process essential for cell migration. These data highlight NDR2’s role in Golgi- and autophagy-regulated migration during starvation. Unlike NDR1, NDR2 is stabilized under starvation and promotes autophagy by regulating LC3 and ATG9A, thereby supporting NSCLC cell proliferation and migration. Routine staining for NDR2 and/or ATG9 could aid in diagnosing NSCLC with high migratory potential.

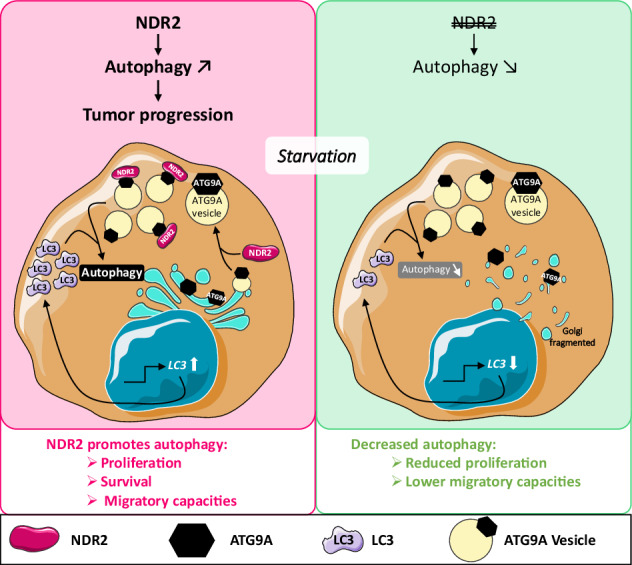

## Introduction

Macroautophagy (hereafter autophagy) is a conserved lysosomal degradation process that maintains cellular homeostasis by removing damaged organelles and proteins [1, 2]. Its dysregulation is linked to cancer [3]. In tumors, autophagy suppresses initiation by clearing defective components and inducing cell death [4, 5], yet under stress (e.g., hypoxia, matrix detachment), it promotes tumor growth and metastasis [6–9].

Non-small cell lung cancer (NSCLC), representing approximately 85% of lung cancer cases, exhibits elevated basal autophagy that supports tumor growth and survival [10–12]. NSCLC is characterized by activation of nuclear Dbf2-related kinase 2 (NDR2) and the transcriptional coactivator YAP-1, both key components of the Hippo pathway [13, 14], which promotes tumor progression by enhancing cell migration and brain metastasis formation [14, 15].

The involvement of NDR2 in autophagy has been established in neurodegenerative diseases and osteoclastogenesis [16, 17], but remains poorly characterized in cancer. Notably, while NDR2 regulates autophagy in breast cancer [18, 19], its role in NSCLC remains unexplored. However, the Drosophila homolog of NDR1/2 interacts with autophagy-related protein 8 (ATG8), the mammalian LC3 equivalent [20]. Additionally, the mammalian autophagy protein ATG9A harbors a phosphorylation motif targeted by NDR2 [21, 22]. Since ATG9A is critical for early autophagosome biogenesis—delivering lipids and proteins to phagophore initiation sites via vesicular trafficking from the Golgi and endosomes [23–30]—it is plausible that NDR2 regulates ATG9A localization or activity.

Here, we demonstrate that NDR2, but not NDR1, is expressed in starved human bronchial epithelial tumor cells (HBECs) and promotes autophagy through regulation of LC3 and ATG9A. This NDR2-mediated autophagic activity enhances HBEC proliferation and migration, implicating NDR2 as a key modulator of autophagy in NSCLC progression.

## Results

### Serum deprivation for 24 h reduces NDR1 but not NDR2 in HBECs

HBEC cell lines (H2030, H2030-BrM3, and H1299) at 60–70% confluence (avoiding YAP-1 inhibition due to overconfluence) were cultured without fetal bovine serum (FBS) for 24 hours (deprivation) to induce autophagy [31]. Such starvation reduced cell density (Fig. S1A), but no apoptotic features were observed; rather the decrease was attributable to reduced cell proliferation, as indicated by Ki67 staining (Fig. S1A/B/C). HBECs are thus resistant to 24 h of FBS deprivation even if they proliferate less under these culture conditions.

**Fig. 1:**
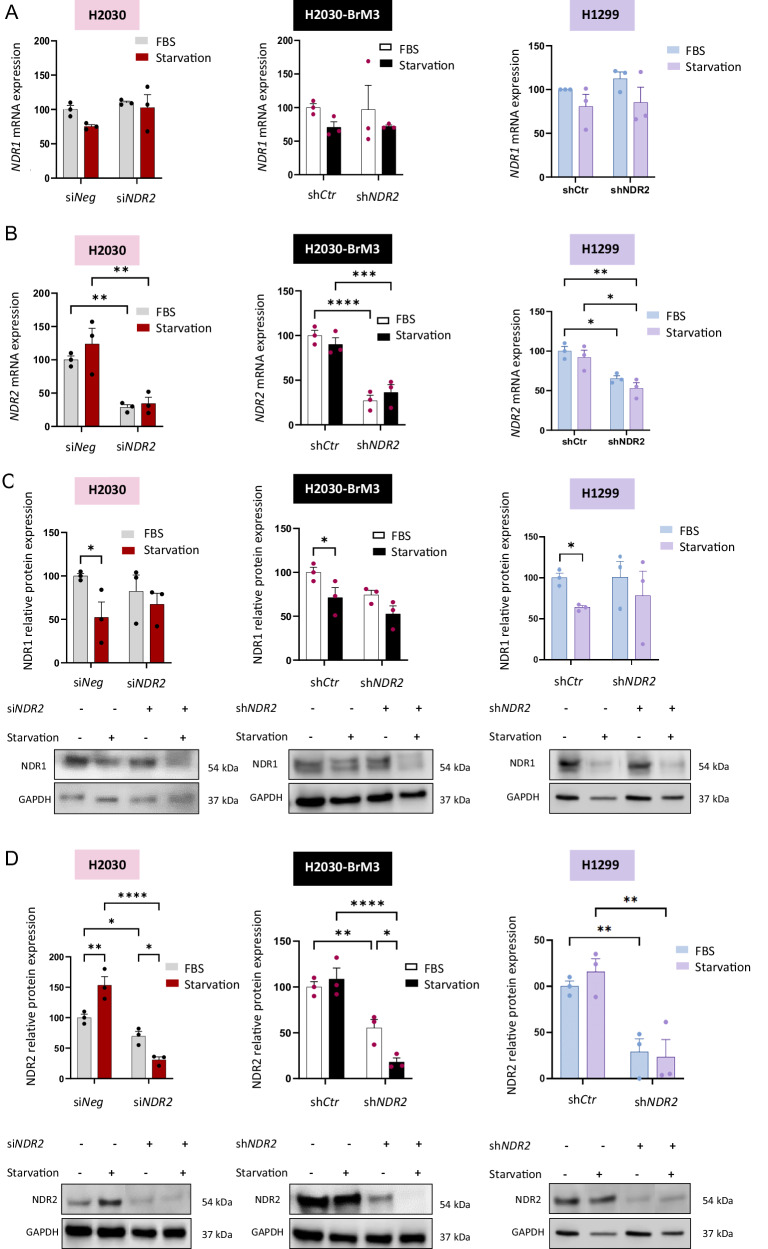
NDR2 is expressed in HBECs grown in the absence of serum for 24 h, unlike NDR1. NDR2 was silenced (si*NDR2* or sh*NDR2*) in H2030, H2030-BrM3 and H1299 cells, which were then deprived of FBS for 24 h; control cells were transfected with si*Neg* or sh*Ctr*. **A** *NDR1* mRNA expression. **B** *NDR2* mRNA expression. **C** NDR1 protein blot and quantification. (**D**) NDR2 protein blot and quantification. The cells were arrested at 80% confluence. Two-way ANOVA followed by Tukey’s post hoc test were used to analyze the data, means ± SEMs, N = 3, *p < 0.05, **p < 0.01, ***p < 0.001 and ****p < 0.0001.

To assess the effect of starvation on NDR1 and NDR2, we measured their mRNA and protein expression levels. Starvation did not alter NDR1 or NDR2 mRNA levels (Fig. 1A/B) but reduced NDR1 protein expression across all cell lines (Fig. 1C). Conversely, NDR2 protein levels increased depending on the cell line (Fig. 1D). Next, we depleted NDR2 expression in HBECs using siRNA or shRNA to determine whether NDR1 compensates for the loss of NDR2 function in HBECs cultured under FBS starvation conditions (Fig. 1B/D). Combined with serum deprivation, NDR2 depletion reduces NDR1 protein levels (Fig. 1C) but not its mRNA expression (Fig. 1A).

We then investigated whether NDR2 functions through the canonical Hippo pathway mechanism, that is, whether it is activated by MST kinases and regulates YAP-1 activity. During FBS starvation of H2030, NDR2 was not activated by the Hippo kinase MST, since FBS starvation silenced MST1 and MST3 but not MST2 (Fig. S2A). Starvation alone did not change YAP-1 nuclear localization but affected target gene expression variably (Fig. S3A/B/C). Silencing NDR2 reduced YAP-1 nuclear localization similarly to starvation (not significant in H2030 cells), and combining NDR2 depletion with starvation did not produce an additive effect (Fig. S3A), suggesting FBS deprivation activates NDR2 via a noncanonical Hippo pathway to regulate YAP-1.

### NDR2 promotes autophagy induction by increasing LC3-II levels in HBECs

**Fig. 2:**
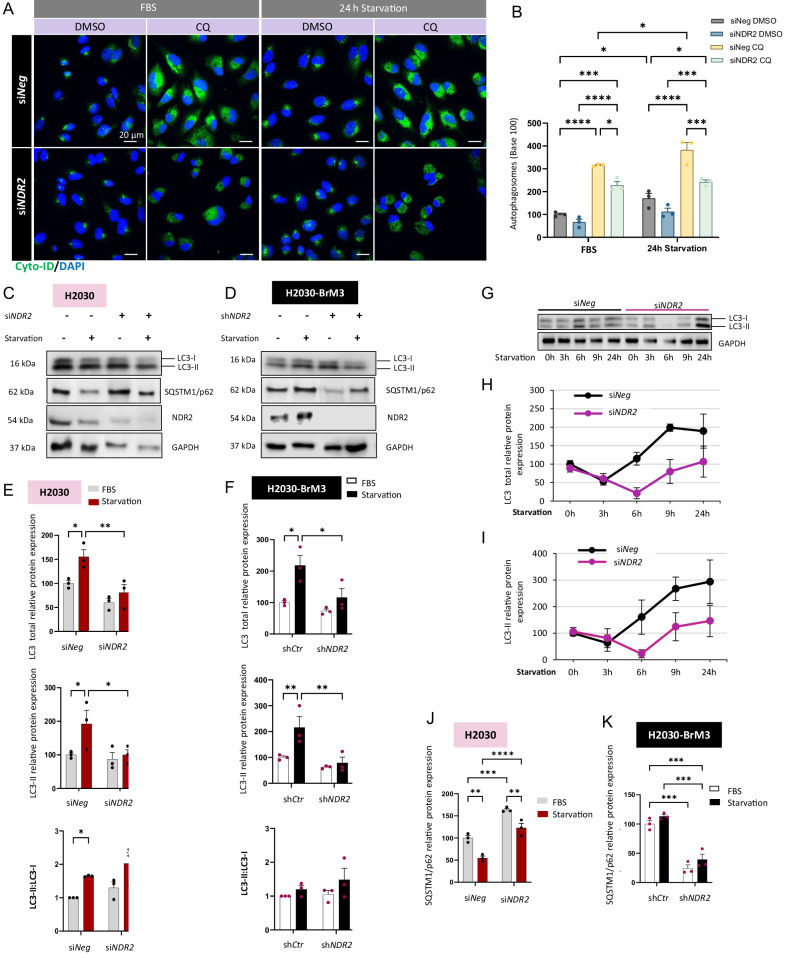
NDR2 promoted autophagosome production in H2030 or H2030-BrM3 cells subjected to serum deprivation for 24 h. **A** H2030 cells were cultured in complete media (FBS) or starvation media (starvation) with or without DMSO or chloroquine (CQ) (10 µM) for 24 h and then treated with Cyto-ID. **B** Quantification of the intensity in (**A**). Blot showing LC3 and NDR2 levels in H2030 (**C**) and H2030-BrM3 (**D**) cells expressing or lacking NDR2 after 24 h of FBS deprivation. **E** Quantification of the blots in (**C**), including total LC3 expression, LC3-II expression and the LC3-II:LC3-I ratio, for H2030 cells. **F** Quantification of the blots in (**D**), including the expression of total LC3, expression of LC3-II and the LC3-II:LC3-I ratio, for H2030-BrM3 cells. **G** Blot showing kinetics of LC3 in H2030 and quantification of LC3 (**H**) and LC3-II (**I**) expression over 24 h of starvation, with or without NDR2 depletion. Quantification of SQSTM1/p62 protein levels in H2030 (**J**) and H2030-BrM3 cells (**K**). Two-way ANOVA followed Tukey’s post hoc test were used to analyze the data, means ± SEMs, N = 3, *p < 0.05, **p < 0.01, ***p < 0.001 and ****p < 0.0001.

We tested whether NDR2 is involved in autophagy in HBECs. Treatment of H2030 cells with chloroquine (CQ, 10 µM) for 24 hours, an inhibitor of autophagosome-lysosome fusion [32], increased autophagosome numbers (Fig. 2A/B). After CQ treatment, cells were subjected to 24 h of FBS starvation, which further increased autophagosome numbers (Fig. 2B). However, NDR2 depletion tended to reduce autophagosome numbers in HBECs under both starvation and non-starvation conditions and significantly lowered them in CQ-treated HBECs (Fig. 2A/B). Notably, the autophagosome increase induced by combined CQ and starvation was absent in NDR2-depleted cells (Fig. 2A/B).

Analysis of LC3, a key marker of autophagosome formation with LC3-II conversion indicating autophagy induction [33], showed decreased mRNA (Fig. S4A) and protein levels (Fig. 2C/D/E/F) upon NDR2 depletion, both under standard conditions and during starvation. The decrease in LC3 expression resulting from NDR2 silencing correlated with fewer LC3 puncta detected via immunofluorescence (Fig. S4B/C), indicating that the loss of NDR2 influenced the number of autophagosomes under serum deprivation conditions. In addition, NDR2 depletion decreased the protein expression of LC3 and LC3-II but only under starvation conditions (Fig. 2C/D/E/F). However, no difference was observed in LC3-II**:**LC3-I ratios (Fig. 2 E/F), suggesting that NDR2 was not involved in LC3 lipidation. During the 24 h starvation period, LC3 and LC3-II expression transiently decreased within the first 3 h, followed by an increase indicating autophagy activation (Fig. 2G/H/I). In NDR2-depleted cells, this increase occurred only after 6 h and remained lower than in controls (Fig. 2G/H/I). These findings suggest that NDR2 contributed to the regulation of autophagic mechanisms, by modulating LC3 expression during starvation (Figs. 2E/F/G/H/I and S4A/B/C).

NDR2 seemed more involved in serum deprivation-induced autophagy than in basal autophagy in HBECs, unlike NDR1, whose loss did not affect LC3 expression during serum starvation (Fig. S4D/E). The LC3 expression induced by NDR2 under starvation conditions may occur through the indirect activation of YAP1 by NDR2 (Fig. S3D/E).

SQSTM1/p62, a cargo degradation mediator whose levels decrease during autophagy [34, 35], showed increased mRNA expression following deprivation in HBECs (Fig. S4F). NDR2 depletion did not influence *SQSTM1/p62* mRNA levels in H2030 cells, but decreased these levels in H2030-BrM3 cells (Fig. S4F). SQSTM1/p62 protein levels decreased in H2030 cells during deprivation, consistent with autophagosome degradation, but increased upon NDR2 silencing regardless of FBS presence (Fig. 2J). In H2030-BrM3 cells, SQSTM1/p62 levels were similar under serum and starvation conditions but NDR2 silencing decreased protein expression in both conditions (Fig. 2K), strengthening the hypothesis that NDR2 is involved in autophagy in HBECs.

To assess whether NDR2 influences autophagy through transcription factor EB (TFEB), a key regulator of autophagy-related genes, we measured the expression of selected TFEB target genes. In HBECs, we observed no variation in the expression of TFEB target genes (*ATG3, ATG9, LAMP1, BECN1*) attributable to NDR2 (Fig. S4G/H).

### NDR2 prevents the accumulation of lysosomes and aggregates in HBECs

**Fig. 3:**
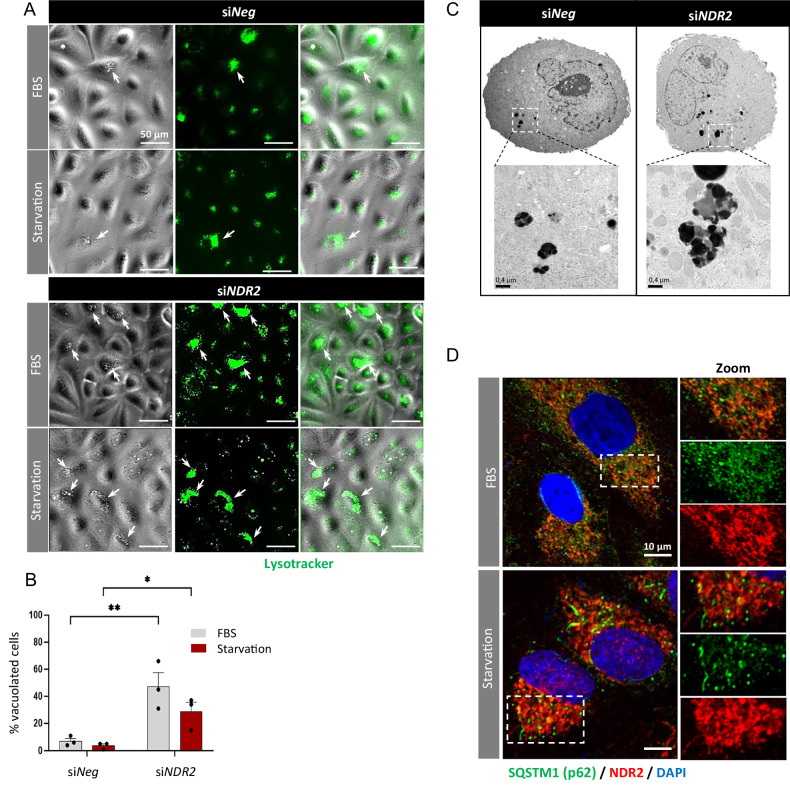
NDR2 silencing induced the accumulation of lysosomes and aggregates in H2030 cells grown in the absence of serum for 24 h. **A** Phase contrast and fluorescence images of H2030 cells expressing or lacking NDR2 showing lysosome labeling with LysoTracker (green). **B** Quantification of vacuolated cells after transfection with si*NDR2* and FBS deprivation for 24 h. **C** TEM images of H2030 cells transfected with either si*Neg* or si*NDR2* showing the accumulation of hyperdense structures. **D** Immunostaining for NDR2 and SQSTM1/p62 in H2030 cells upon serum deprivation for 24 h. Two-way ANOVA followed by Tukey’s post hoc test were used to analyze the data, means ± SEMs, N = 3, **p < 0.05, **p < 0.01, ***p < 0.001 and ****p < 0.0001.

Following NDR2 silencing, an increase number of cells with large pericentriolar vacuolar structures was observed (Fig. 3A/B). With LysoTracker labeling, these structures were identified as primarily lysosomes (Fig. 3A). As observed with TEM, NDR2 depletion in HBECs induced the accumulation of much larger hyperdense structures than did NDR2 depletion in control (si*Neg*) cells (Fig. 3C), suggesting impaired cargo degradation and disrupted lysosomal trafficking or autophagosome fusion.

NDR2 kinase is essential for autophagy but does not directly interact with autophagosomes. Labeling of NDR2 and SQSTM1/p62 in H2030 cells revealed their proximity but no strict colocalization (Fig. 3D).

### The shift in ATG9A subcellular localization in HBECs depends on NDR2

**Fig. 4:**
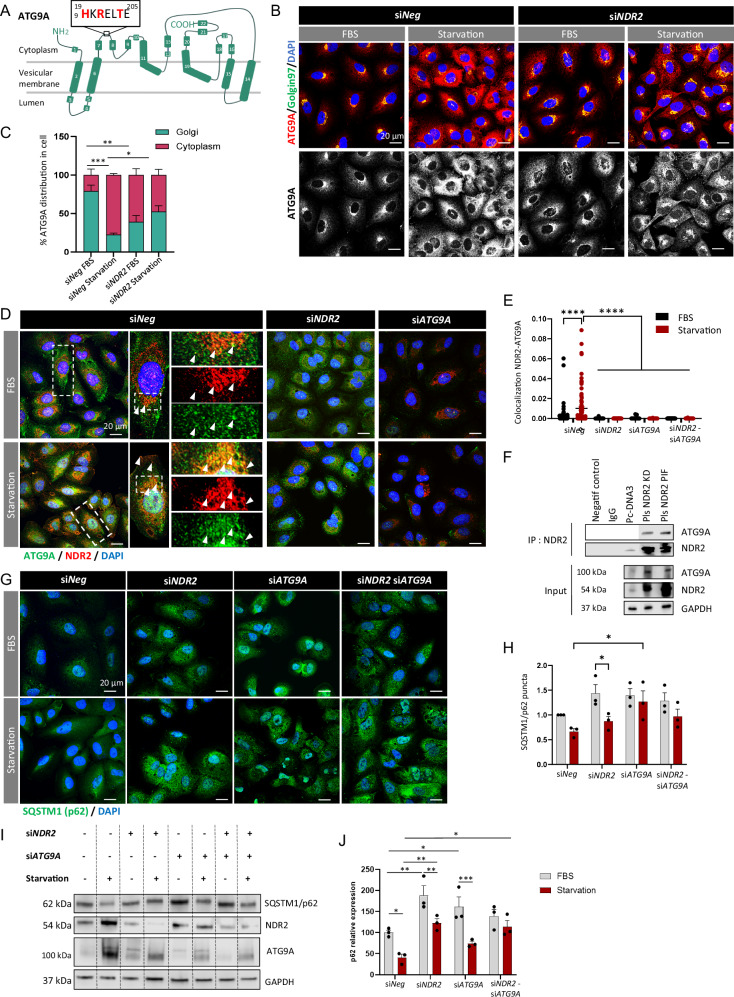
NDR2 governs the subcellular localization of ATG9A in H2030 cells. **A** Structural diagram of ATG9A. ATG9A possesses the consensus NDR2 phosphorylation motif (HXRXXT) in its cytoplasmic domain. Immunolabeling (**B**) and quantification of ATG9A localization (**C**) upon FBS deprivation for 24 h, with or without NDR2 silencing, in H2030 cells. **D** Colocalization of NDR2 and ATG9A immunolabeling in H2030 cells under normal or serum-deprived conditions and transfection of siNDR2 or siATG9A. **E** Quantification of NDR2-ATG9A colocalization. **F** Co-immunoprecipitation of ATG9A with NDR2 in H2030 cells, either non-transfected or transfected with a plasmid expressing a hyperactive NDR2 kinase (PIF), a kinase-dead variant (KD), or an empty pcDNA3 vector. NDR2 or/and ATG9A (siNDR2 or siATG9A) silenced or control (siNeg) H2030 cells were subjected to 24 h of serum deprivation or control culture. Immunolabeling (**G**) and quantification of SQSTM1/p62 puncta (**H**). **I** SQSTM1/p62 protein blot. **J** SQSTM1/p62 protein quantification. Two-way ANOVA followed by Tukey’s post hoc test were used to analyze the data, means ± SEMs, *N* = 3, *p < 0.05, **p < 0.01, ***p < 0.001 and ****p < 0.0001.

ATG9A is a transmembrane protein found in the trans-Golgi network (TGN), endosomes, and autophagosomes [23, 25] (Fig. 4A). Under serum starvation, ATG9A-positive vesicles fuse with phagophores, enabling their expansion into autophagosomes [23, 25]. NDR2 silencing changed the subcellular localization of ATG9A under normal conditions in HBECs (Figs. 4B/C and S5A/B). ATG9A translocated from the TGN to the cytoplasm in HBECs after FBS starvation (Fig. 4B/C), consistent with the increased number of autophagosomes observed under these conditions (Fig. 2). Serum-starvation-induced localization of ATG9A appears to require the presence of NDR2: in HBECs lacking NDR2, ATG9A is evenly distributed between the Golgi and the cytoplasm, whether cells are cultured in complete or serum-free medium (Figs. 4B/C and S5A/B). A colocalization of NDR2 with ATG9A was observed in H2030 cells with complete and starved medium, as in other HBECs (Fig. S5C/D), but not in cells transfected with siNDR2 or siATG9A as controls (Fig. 4D/E). Thus, NDR2 may contribute to ATG9A vesicle trafficking and phagophore biogenesis. This hypothesis was strengthened the presence of the NDR2 consensus phosphorylation motif (HxRxxS/T) [36] in the ATG9A sequence (Fig. 4A). Immunoprecipitation revealed an interaction between NDR2 and ATG9A consistent with substrate-enzyme relationship, suggesting that NDR2 may phosphorylate ATG9A to facilitate its trafficking (Fig. 4F).

NDR2 and ATG9A co-depletion increased SQSTM1/p62 levels and puncta under both FBS and starvation, without additive effects, suggesting an involvement in common mechanism in autophagic processes (Fig. 4G/H/I/J).

**Fig. 5:**
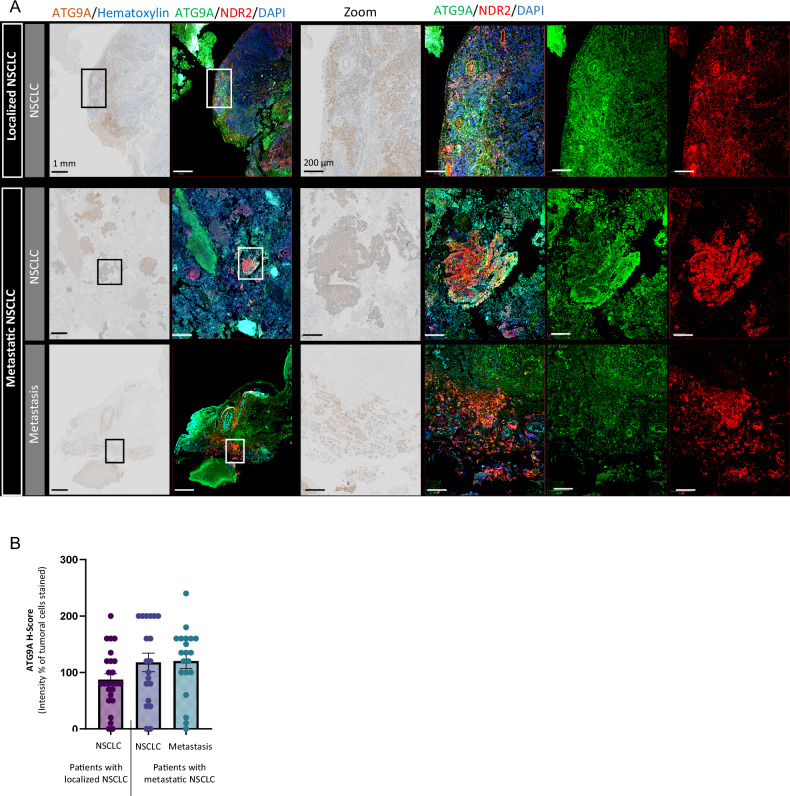
ATG9A is expressed in NSCLC and colocalizes with NDR2. **A** We immunostained a retrospective population of patients operated on a non-metastatic NSCLC (n = 25) or metastatic NSCLC (n = 20) for whom both the primitive tumor and the brain metastasis (BM) were available, with ATG9A (1:100) and we costained in fluorescence with NDR2 (1:200) and ATG9A (1:100). **B** Data are presented as the mean ± SEM of an IHC score, calculated by multiplying the staining intensity (0–3) by the distribution percentage (0–100%) and labeling localization.

This phenomenon may also occur in NSCLC tumors. We previously demonstrated that NDR2 is more highly expressed in metastatic NSCLC [14]. In the same patient cohort (25 cases of localized NSCLC and 20 cases of metastatic NSCLC), ATG9A expression also showed a tendency to be higher, although not significantly, in metastatic samples (Fig. 5). Consistent with our in vitro observations, the colocalization of NDR2 and ATG9A reveals a close spatial association between these proteins (Fig. 5A), suggesting a potential functional interaction that may promote autophagy in NSCLC.

### NDR2 and ATG9A maintain the integrity of the Golgi apparatus

**Fig. 6:**
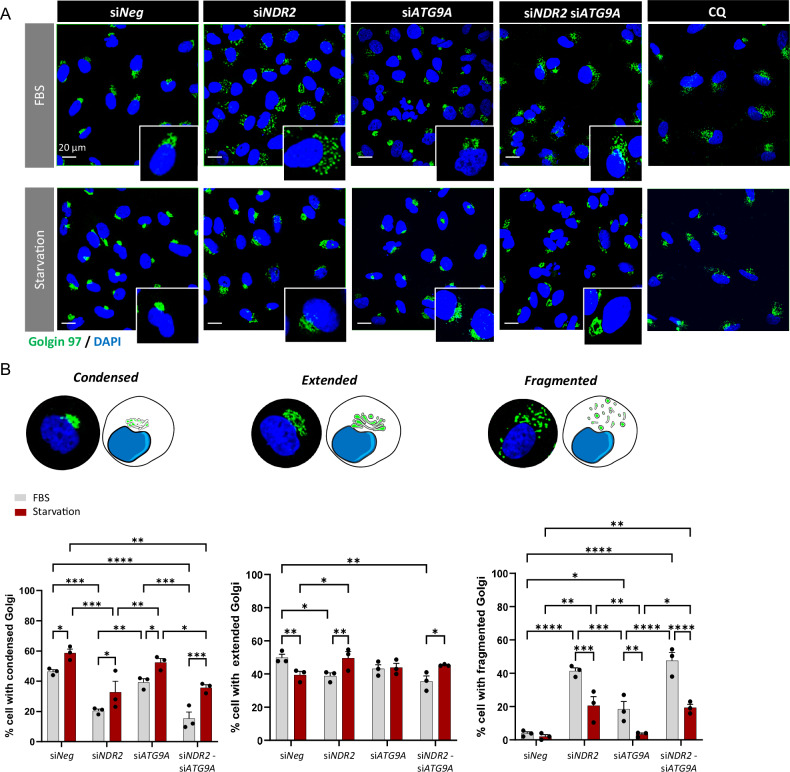
NDR2 and ATG9A prevented the fragmentation of the Golgi apparatus in H2030 cells. **A** Immunolabeling of the Golgi with golgin-97 (green) in the H2030 cell line. **B** Diagram of the different classes of the Golgi identified in (**A**) (condensed, extended and fragmented) and quantification. A minimum of 150 cells were counted per experiment. Two-way ANOVA followed by Tukey’s post hoc test were used to analyze the data, means ± SEMs, N = 3, *p < 0.05, **p < 0.01 and ***p < 0.001.

NDR2 depletion disrupted Golgi structure in H2030 cells (Fig. 6A). We categorized Golgi architecture into three distinct states: condensed, extended, or fragmented (Fig. 6B). The Golgi apparatus was condensed or extended in control condition (Fig. 6A/B). Under both serum deprivation and non-deprivation conditions, NDR2 silencing increased Golgi fragmentation (Fig. 6A/B). ATG9A depletion also caused Golgi fragmentation but to a lesser extent, with no additive effect seen during combined NDR2-ATG9A silencing. In contrast, FBS deprivation alone led to Golgi condensation (Fig. 6B). Golgi fragmentation was also observed with CQ treatment, suggesting that defects in autophagy may underlie fragmentation seen with NDR2 and/or ATG9A loss. (Fig. 6).

### NDR2-ATG9A control H2030 cells migration properties

**Fig. 7:**
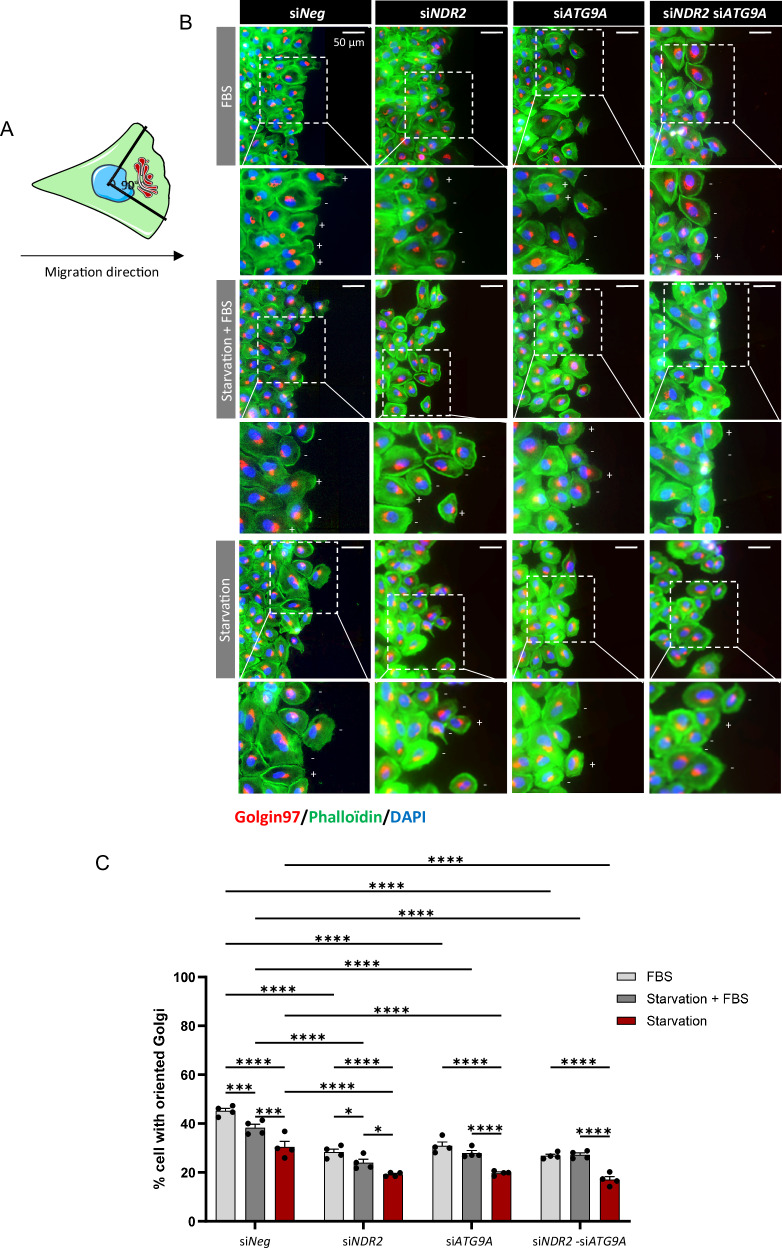
NDR2 and ATG9A are required for the reorientation of the Golgi apparatus during migration. **A** Schematic representation of the disposition of the Golgi in migrating cells. **B** Illustration of the position of the Golgi (red) during migration (t6 h), (+) indicates cells with Golgi apparatus oriented toward the wound, (–) indicates cells with non-oriented Golgi. **C** Quantification of cells with the Golgi oriented toward the migration front during serum deprivation with or without the loss of NDR2 or ATG9A. Two-way ANOVA followed by Tukey’s post hoc test were used to analyze the data, means ± SEMs, N = 4, *p < 0.05, **p < 0.01, ***p < 0.001 and ****p < 0.0001.

**Fig. 8:**
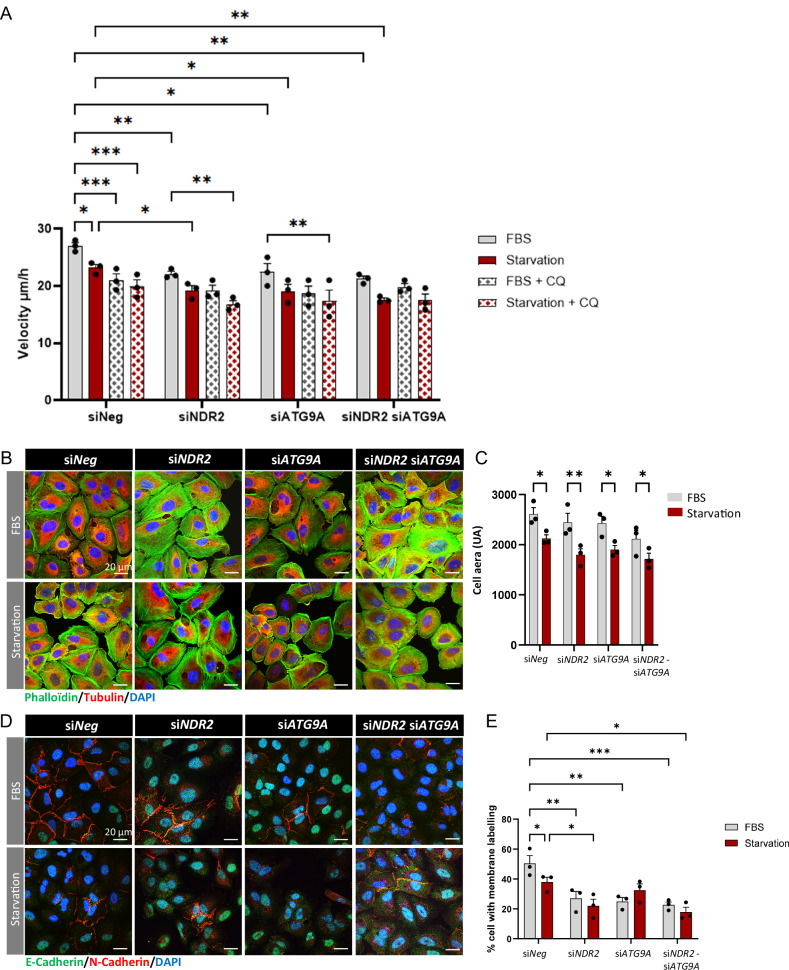
FBS deprivation and the loss of NDR2 or ATG9A decreased the migration capacity of H2030 cells. **A** Quantification of the wound-healing assay with H2030 cells with or without NDR2 and/or ATG9A silencing in full media or starvation media and with or without CQ. **B** Immunolabeling for phalloidin (green) and tubulin (red). **C** Quantification of the cell area using phalloidin labeling (**B**). **D** Immunolabeling of E-cadherin (green) and N-cadherin (red). **E** Quantification of the number of cells with N-cadherin membrane labelling and analysis via two-way ANOVA followed by Tukey’s post hoc test, means ± SEMs, N = 3, *p < 0.05, **p < 0.01, ***p < 0.001 and ****p < 0.0001.

A wound-healing assay was conducted after 24 h of serum deprivation or culture in complete medium, followed by cell migration under the same respective conditions. Six hours after wound healing, we quantified the number of HBECs exhibiting Golgi orientation toward the wound edge, indicative of directional migration [37]. Golgi orientation was defined as the apparatus positioned within a 90° angle from the nuclear center toward the wound [37, 38] (Fig. 7A). During starvation, the reorientation of the Golgi toward the migration front decreased in HBECs (Figs. 7B/C, S6A/B, S7A/B), which coincided with the decrease in the cell migration velocity (Fig. 8A and S8). NDR2 or ATG9A silencing decreased the number of cells with the Golgi apparatus positioned toward the migration front under all conditions (Fig. 7B/C), consistent with the decrease in migration speed observed in the presence or absence of serum for H2030 (Fig. 8A).

Although MST3 was not expressed under starvation conditions (Fig. S2), its involvement in NDR2 activation and cell migration [39–41] led us to investigate this Golgi-associated kinase. Despite its stronger perinuclear/Golgi localization in metastatic versus localized NSCLC samples (Fig. S2B), MST3 did not affect Golgi structure or localization in H2030 cell (Fig. S2C/D). NDR2 effects on the Golgi was independent of its activation by MST3.

FBS deprivation before and after wounding reduced migration speed and shifted cells from collective to individual migration in HBECs (Figs. S6A/B, S7A/B, S8). Cells subjected to starvation followed by FBS replenishment (Starvation+FBS) migrated at similar speeds to those maintained in complete medium, except for H1299 cells, which migrated faster. However, this increase was absent upon inhibition of NDR2 (Fig. S7), which reduces filopodia formation by modifying the actin cytoskeleton without affecting vimentin or tubulin organization (Fig. S7D/E/F). No cytoskeletal changes were observed in other HBECs, while cell area decreased under serum deprivation (Figs. 8B/C and S6D).

NDR2 depletion reduced H2030 cell migration regardless of FBS availability (Fig. 8A). ATG9A silencing impaired migration only under FBS deprivation, while combined NDR2-ATG9A depletion decreased migration in all conditions (Figs. 8A and S8). Inhibition of autophagy by CQ reduced the migration rate of H2030 cells both in the presence and absence of FBS, indicating that autophagy contributed to the migratory capacity of these cells. NDR2 depletion alone results in a decrease comparable to that observed in siNeg cells treated with CQ. Moreover, CQ treatment of NDR2-depleted cells did not further enhance this effect. Similar results are observed following ATG9A depletion, either alone or in combination with NDR2 depletion (Fig. 8A). These findings suggested that NDR2 and ATG9A jointly promote cell migration under both nutrient-deprived and FBS-supplemented conditions, likely through the regulation of autophagy.

NDR2 and/or ATG9A loss also reduced membrane-associated N-cadherin, which accumulated perinuclear region (Fig. 8D/E).

## Discussion

Consistent with the high basal autophagy in NSCLCs [42] and previous findings [43], we demonstrate here that HBECs tolerate 24 h of serum starvation. Moreover, we reveal that NDR1 is not involved in autophagy in HBECs and highlight a novel role for NDR2 kinase in bronchial carcinogenesis, specifically in autophagy initiation and autophagy-dependent migration. Indeed, while NDR1 regulates autophagosome formation in other models [42, 44–47], its expression is silenced in serum-starved HBECs, unlike NDR2. Moreover, NDR1 does not compensate for NDR2 silencing by siRNA or shRNA under these conditions. Thus, NDR2 appears to be the key NDR kinase controlling autophagy in HBECs. This supports previous reports [14, 22, 47, 48] indicating that NDR1 and NDR2 have non-redundant, and possibly opposing roles. Additional experiments are required to clarify: (i) the mechanism responsible for NDR1 degradation without affecting NDR2 in serum-starved HBECs, and (ii) how NDR2 activity is increased under these conditions. One possible explanation for NDR1 silencing is SOCS2, a starvation-induced E3 ligase that ubiquitinates NDR1 and triggers its degradation in mouse fibroblasts [48, 49]. Regarding NDR2 expression in serum-starved HBECs, our data indicate that its regulation is independent of Hippo MST kinases, as both MST1 and the NDR2-specific MST3 are then silenced. Instead, NDR2 may be activated *via* noncanonical pathways, such as MAP4K4, which promotes autophagy through SOX6 [50], or through SIRT1 [51], a key autophagy regulator [52].

Here, we show that once activated, NDR2 regulates autophagy through an indirect mechanism involving LC3 and ATG9A, further underscoring its oncogenic role. Similar to other NDR kinases (e.g., LATS1/2, NDR1), NDR2 regulates all proteins containing the HxRxxS/T motif [53], including YAP1 and TAZ, thereby influencing cell proliferation and survival [53], as well as additional substrates—not all yet identified—that support its diverse roles in both healthy and tumor cells [21, 54–61], particularly during hypoxia and metastatic dissemination in NSCLC. NDR2 is essential for autophagosome formation in HBECs, as its silencing reduces autophagosome numbers and promotes aggregate formation. NDR1/2 knockout lowers LC3 levels in neurons, but NDR2’s specific role in LC3 regulation remains unclear, unlike NDR1 [16]. In this study, we observed a decrease in LC3 expression in serum-starved HBECs following the depletion of NDR2, which could be one of the reasons for the observed reduction in autophagosome formation in HBECs. The regulation of LC3 by NDR2 does not depend on TFEB activity but could be mediated by YAP-1. Indeed, LC3 can also be transcribed by YAP-1 [62] and c-JUN [63], two transcription factors whose activity is promoted by NDR2 in hypoxic NSCLC cells [14]. YAP-1 activity decreased upon NDR2 loss in both serum-deprived and non-serum-deprived HBECs. The use of siYAP-1 resulted in a decrease in LC3 mRNA expression under deprivation conditions, with no additive effect observed when NDR2 and YAP-1 were simultaneously depleted, suggesting a novel regulatory axis of LC3 expression *via* NDR2-YAP-1. NDR2 also appears to transcriptionally regulate SQSTM1/p62 in a cell line-dependent manner, as its depletion reduces SQSTM1/p62 mRNA levels—but only in H2030-BrM3 cells. This finding supports the notion that NDR2 regulates a transcription factor involved in autophagy.

NDR2 regulates the subcellular localization of ATG9A, which plays a crucial role in early phagophore formation. Loss of NDR2 leads to ATG9A maldistribution and Golgi fragmentation. Golgi disruption impairs autophagy by affecting lipid and protein supply, as well as disrupting ATG9A vesicle trafficking [21, 64–66]. This observation aligns with Rosianu et al., who reported ATG9A peripheral localization and impaired autophagy in neurons following NDR1/2 knockout in mice [16]. Another mechanism may exist, as ATG9A contains an NDR2 phosphorylation motif. NDR2 could directly interact with ATG9A to promote its trafficking, potentially through the actin network [67], with which NDR2 also interacts, and via adaptor protein (AP) complexes, thereby enhancing autophagy [68]. Additional experiments are necessary to elucidate the precise mechanisms by which NDR2 may facilitate ATG9A vesicular trafficking and autophagosome biogenesis.

Autophagy influences cell migration in a context-dependent manner, either inhibiting it by regulating adhesions [69, 70] or promoting it via adhesion disassembly and EMT notably in NSCLC [71, 72]. Here, we showed that NDR2 and ATG9A are both involved in the localization of the Golgi apparatus in HBECs. NDR2 and/or ATG9A silencing disrupts the reorientation of the Golgi apparatus toward the leading edge of the cell in migration assays. Migrating cells establish polarity by reorienting the Golgi toward the leading edge, enabling targeted secretion that promotes cancer cell migration, invasion, and metastasis [37, 73]. Improper Golgi reorientation disrupts the accumulation of cytoskeletal elements at the wound site and impairs the recycling of key migration components [74, 75]. Under these conditions, the decrease in Golgi reorientation upon the depletion of NDR2 and/or ATG9A and the decrease in autophagy under these same conditions could impact the renewal and recycling of focal adhesion plaques and integrins and, consequently, cell migration. In glioblastoma, ATG9A promotes integrin trafficking to enable tumor cell migration [76]. Similarly, NDR2 may facilitate integrin trafficking *via* ATG9A in HBECs, consistent with its reported role in integrin transport during neuronal development [60, 61].

In conclusion, these data indicate that NDR2 has a novel role in autophagy in NSCLC cells under prolonged serum starvation by regulating LC3 and ATG9A. Additionally, the NDR2–ATG9A pathway enhances NSCLC cell migration during starvation. Further studies will be required to explore how the NDR2-ATG9A relationship interfaces with that of NDR2 and ULK1 [17, 18, 25, 27] during the autophagic process in healthy or tumor cells.

## Materials and methods

### Patients

We selected a retrospective cohort of 45 patients who underwent surgery for nonmetastatic NSCLC (n = 25) or metastatic NSCLC (n = 20) at Caen University Hospital between December 2009 and December 2019, with both primary tumor and brain metastasis samples available. Among the 25 localized NSCLC patients, 17 were men and 8 women, with a mean age of 71 years (range 54–86). Among the 20 metastatic NSCLC patients, 15 were men and 5 women, with a mean age of 67 years (range 40–82). All patients gave informed consent, and the study was approved by the institutional ethics committee, in accordance with French law (North-west-Committee-for-Persons-Protection-III No. DC-2008-588).

### Cell culture transfection and treatments

The H1299 (CRL-5803) and H2030 (CRL-5914) lung adenocarcinoma cell lines were obtained from ATCC, while the brain-tropic H2030-BrM3 line was kindly provided by Dr. Joan Massagué (MSKCC, USA). All cells were cultured in high-glucose DMEM (4.5 g/L) supplemented with 10% heat-inactivated FBS, 100 U/mL penicillin, 100 μg/mL streptomycin, and 2 mM L-glutamine (Gibco). Stable shRNA-depleted lines for NDR1 and NDR2 (shNDR1, shNDR2), along with a control line (shCtr), were used for H1299 and H2030-BrM3, as described [15]. siRNA duplexes (Eurogentec®, Table S1) or plasmids (kindly provided by Dr. Alexander Hergovich, University College London) were transfected using JetPrime (Polyplus, 101000046) following the manufacturer’s protocol at 40% cell confluence. Transfection efficiency was routinely confirmed by RT‒PCR and/or Western blot.

For FBS starvation, cells were cultured for 24 h at 37 °C and 5% CO₂ in low-glucose DMEM (1000 mg/L) without FBS, supplemented with antibiotics and antimycotics.

For some experiments, chloroquine (CQ) was added at 10 µM to the culture medium for up to 24 hours.

The cells were regularly tested for mycoplasma contamination.

### Preparation of RNA and RT-PCR

Total RNA was extracted using the ReliaPrep™ RNA Miniprep System (Promega) per the manufacturer’s protocol. Reverse transcription was performed on 250 ng RNA with random primers and 200 IU M-MLV reverse transcriptase at 37 °C for 90 min, followed by inactivation at 70 °C for 5 min (Mastercycler, Eppendorf®). cDNA was diluted 1:10 and used for qPCR on an Mx3005P system (Agilent Technologies) with 5 pmol primers and iQ™ SYBR Green Supermix (Bio-Rad). S16 was the internal control; positive and no-RT controls were included. Gene expression was quantified using the ΔΔCt method. The primers used are listed in Table S2.

### Antibodies, immunofluorescence staining, immunoblotting, Immunoprecipitation

The antibodies used are listed in Table S3.

For immunofluorescence, 2 × 10⁴ cells were seeded on coverslips in 24-well plates, washed with PBS, fixed with 4% paraformaldehyde (20 min, 37 °C), and permeabilized with methanol (10 min, –20 °C). After blocking with 4% BSA in PBS for 1 hour, cells were incubated overnight at 4 °C with primary antibodies. Alexa-488 or Alexa-555 secondary antibodies (ThermoFisher Scientific) were applied for 1 h at room temperature. Nuclei were counterstained with DAPI (Fluoromount-GTM, Invitrogen). Imaging was performed on an Olympus FluoView FV1000 confocal microscope (60×, NA 1.20).

For immunoblotting, whole-cell extracts were prepared as described [14]. Proteins were separated by SDS-PAGE and detected with primary antibodies (1:1000) in TBS-T (0.1% Tween-20), HRP-conjugated secondary antibodies, and Clarity Western ECL (Bio-Rad). Signals were captured with an Odyssey Fc imager (Li-COR®) and quantified using ImageJ. GAPDH was used as a loading control.

Immunoprecipitation was carried out on extracts from T75-flask cultures using the Pierce Co-IP Kit (Thermo Scientific, 26149) following the manufacturer’s protocol.

Immunohistochemistry (IHC) was performed on 3 µm FFPE sections using the Discovery Ultra Ventana analyzer. After deparaffinization and antigen retrieval (Tris-EDTA buffer, pH 8.2), sections were incubated with anti-NDR2 (1:200), anti-ATG9 (1:100), and anti-MST3 (1:200). Denaturation was done with Ultra CC2 (Ventana, #950-223, 8 min, 100°C). For co-staining, HRP-conjugated secondary antibodies were detected using DISC. CY5 and FITC Kits (Ventana, #253-4928, #253-4881). Nuclei were counterstained with DAPI (Ventana, #760-4196).

### Autophagy assay

The CYTO-ID® Autophagy Detection Kit (Enzo Life Sciences, ENZ-51031-0050) was used following the manufacturer’s instructions. Cells on glass coverslips were treated with 10 µM CQ for 24 hours prior to CYTO-ID staining. Images were acquired with an Olympus FluoView FV1000 confocal microscope and analyzed using ImageJ.

### LysoTracker staining

LysoTracker™ Green DND-26 (50 nM; Cell Signaling Technology, 8783) was added to the culture medium. Live-cell imaging was performed immediately using a Leica DMi8 inverted microscope in a temperature- and CO₂-controlled chamber (37 °C, 5% CO₂). Images from three fields were captured with Metamorph 7.8.13.0 and analyzed using ImageJ.

### Wound-healing assay

Transfected cells were seeded on collagen I-coated plates or collagen IV-coated plates, grown to confluence, and treated with mitomycin C (1 μg/mL) for 12 h to inhibit proliferation. Cells were then incubated in either full media or starvation media, with or without CQ at 10 µM. A scratch was made with a P-10 pipette tip (0 h), and migration was monitored for 20 h. Images were captured every 15 min using a ZEISS AXIO Observer 7 microscope equipped with a motorized stage, temperature-controlled chamber (37 °C, 5% CO₂, PECON) and a Hamamatsu ORCA FLASH 4 camera. Cell movements were tracked with a custom Python script using Cellpose and Trackpy libraries.

### Statistical analysis

Data are shown as means ± SEM from three independent experiments. Statistical significance (p < 0.05) was assessed using two-tailed Student’s t-test or two-way ANOVA with Tukey’s post hoc test (the normality of the data distribution was assessed and the variance was similar between the groups that are being statistically compared) via GraphPad Prism 4 (San Diego, CA, USA).

## Supplementary information

Figues S1_S8

Tables S1_S3

Original Data

## Data Availability

All data supporting the findings of this study are available within the paper and its Supplementary Information. The datasets used and analyzed during the current study are available from the corresponding authors upon reasonable request.

## References

[1] Mizushima N, Komatsu M. Autophagy: renovation of Cells and Tissues. Cell. 2011;147:728–41. 10.1016/j.cell.2011.10.026. 22078875 10.1016/j.cell.2011.10.026

[2] Zhen Y, Stenmark H. Autophagosome biogenesis. Cells. 2023;12:668. 10.3390/cells12040668. 36831335 10.3390/cells12040668 PMC9954227

[3] Levine B, Kroemer G. Autophagy in the pathogenesis of disease. Cell. 2008;132:27–42. 10.1016/j.cell.2007.12.018. 18191218 10.1016/j.cell.2007.12.018 PMC2696814

[4] Russell R, Guan K-L. The multifaceted role of autophagy in cancer. EMBO J. 2022;41:e110031. 10.15252/embj.2021110031. 35535466 10.15252/embj.2021110031 PMC9251852

[5] Patergnani S, Missiroli S, Morciano G, Perrone M, Mantovani C, Anania A, et al. Understanding the role of autophagy in cancer formation and progression is a real opportunity to treat and cure human cancers. Cancers. 2021;13:5622. 10.3390/cancers13225622. 34830777 10.3390/cancers13225622 PMC8616104

[6] Peng Y-F, Shi Y-H, Shen Y-H, Ding Z-B, Ke A-W, Zhou J, et al. Promoting colonization in metastatic HCC cells by modulation of autophagy. PLoS ONE. 2013;8:e74407. 10.1371/journal.pone.0074407. 24058558 10.1371/journal.pone.0074407 PMC3772859

[7] Mowers E, Sharifi M, Macleod K. Functions of autophagy in the tumor microenvironment and cancer metastasis. FEBS J. 2017;285:1751–66. 10.1111/febs.14388. 10.1111/febs.14388 PMC5992019 29356327

[8] Kroemer G, Mariño G, Levine B. Autophagy and the integrated stress response. Mol Cell. 2010;40:280–93. 10.1016/j.molcel.2010.09.023. 20965422 10.1016/j.molcel.2010.09.023 PMC3127250

[9] Fung C, Lock R, Gao S, Salas H, Debnath J. Induction of autophagy during extracellular matrix detachment promotes cell survival. Mol Biol Cell. 2008;19:797–806. 10.1091/mbc.E07-10-1092. 18094039 10.1091/mbc.E07-10-1092 PMC2262959

[10] Guo J, Xia B, White E. Autophagy-mediated tumor promotion. Cell. 2013;155:1216–9. 10.1016/j.cell.2013.11.019. 24315093 10.1016/j.cell.2013.11.019 PMC3987898

[11] Guo J, Teng X, Laddha S, Ma S, Van Nostrand S, Yang Y, et al. Autophagy provides metabolic substrates to maintain energy charge and nucleotide pools in Ras-driven lung cancer cells. Genes Dev. 2016;30:1704–17. 10.1101/gad.283416.116. 27516533 10.1101/gad.283416.116 PMC5002976

[12] Wang D, He J, Huang B, Liu S, Zhu H, Xu T. Emerging role of the hippo pathway in autophagy. Cell Death Dis. 2020;11:880. 10.1038/s41419-020-03069-6. 33082313 10.1038/s41419-020-03069-6 PMC7576599

[13] Dubois F, Keller M, Calvayrac O, Soncin F, Hoa L, Hergovich A, et al. RASSF1A suppresses the invasion and metastatic potential of human non-small cell lung cancer cells by inhibiting YAP activation through the GEF-H1/RhoB pathway. Cancer Res. 2016;76:1627–40. 10.1158/0008-5472.CAN-15-1008. 26759237 10.1158/0008-5472.CAN-15-1008

[14] Levallet J, Biojout T, Bazille C, Douyère M, Dubois F, Leite Ferreira D, et al. Hypoxia-induced activation of NDR2 underlies brain metastases from Non-Small Cell Lung Cancer. Cell Death Dis. 2023;14:823. 10.1038/s41419-023-06345-3. 38092743 10.1038/s41419-023-06345-3 PMC10719310

[15] Keller M, Dubois F, Teulier S, Martin A, Levallet J, Mailler E, et al. NDR2 kinase contributes to cell invasion and cytokinesis defects induced by the inactivation of RASSF1A tumor-suppressor gene in lung cancer cells. J Exp Clin Cancer Res. 2019;38:158. 10.1186/s13046-019-1145-8. 30979377 10.1186/s13046-019-1145-8 PMC6461807

[16] Roşianu F, Mihaylov S, Eder N, Martiniuc A, Claxton S, Flynn HR, et al. Loss of NDR1/2 kinases impairs endomembrane trafficking and autophagy leading to neurodegeneration. Life Sci Alliance. 2022;6:e202201712. 10.26508/lsa.202201712. 36446521 10.26508/lsa.202201712 PMC9711861

[17] Kong X, Shan Z, Zhao Y, Tao S, Chen J, Ji Z, et al. NDR2 is critical for osteoclastogenesis by regulating ULK1-mediated mitophagy. JCI Insight. 2025; 10.1172/jci.insight.180409. 10.1172/jci.insight.180409 PMC11721311 39561008

[18] Yang Y, Zhu Y, Zhou S, Tang P, Xu R, Zhang Y, et al. TRIM27 cooperates with STK38L to inhibit ULK1-mediated autophagy and promote tumorigenesis. EMBO J. 2022;41:e109777. 10.15252/embj.2021109777. 35670107 10.15252/embj.2021109777 PMC9289709

[19] Thorne R, Yang Y, Wu M, Chen S. TRIMming down autophagy in breast cancer. Autophgy. 2022;18:2512–3. 10.1080/15548627.2022.2105557. 10.1080/15548627.2022.2105557 PMC9542414 35897149

[20] Tsapras P, Petridi S, Chan S, Geborys M, Jacomin A-C, Sagona A, et al. Selective autophagy controls innate immune response through a TAK1/TAB2/SH3PX1 axis. Cell Rep. 2022;38:110286. 10.1016/j.celrep.2021.110286. 35081354 10.1016/j.celrep.2021.110286

[21] Holzer E, Martens S, Tulli S. The role of ATG9 vesicles in autophagosome biogenesis. J Mol Biol. 2024;436:168489. 10.1016/j.jmb.2024.168489. 38342428 10.1016/j.jmb.2024.168489

[22] Biojout T, Bergot E, Bernay B, Levallet G, Levallet J. NDR2 Kinase: a review of its physiological role and involvement in carcinogenesis. Int J Biol Macromol. 311:143656. 10.1016/j.ijbiomac.2025.143656 10.1016/j.ijbiomac.2025.143656 40311964

[23] Orsi A, Dooley H, Robinson D, Weston AE, Collinson LM, et Tooze SA. Dynamic and transient interactions of Atg9 with autophagosomes, but not membrane integration, are required for autophagy. Mol Biol Cell. 2012;23:1860–73. 10.1091/mbc.E11-09-0746. 22456507 10.1091/mbc.E11-09-0746 PMC3350551

[24] Imai K, Hao F, Fujita N, Tsuji Y, Oe Y, Araki Y, et al. Atg9A trafficking through the recycling endosomes is required for autophagosome formation. J Cell Sci. 2016;129:3781–91. 10.1242/jcs.196196. 27587839 10.1242/jcs.196196

[25] Zhou C, Ma K, Gao R, Mu C, Chen L, Liu Q, et al. Regulation of mATG9 trafficking by Src- and ULK1-mediated phosphorylation in basal and starvation-induced autophagy. Cell Res. 2017;27:184–201. 10.1038/cr.2016.146. 27934868 10.1038/cr.2016.146 PMC5339848

[26] Noda T. Autophagy in the context of the cellular membrane-trafficking system: the enigma of Atg9 vesicles. Biochem Soc Trans. 2017;45:1323–31. 10.1042/BST20170128. 29150528 10.1042/BST20170128 PMC5730941

[27] Young A, Chan E, Hu X, Köchl R, Crawshaw S, High S, et al. Starvation and ULK1-dependent cycling of mammalian Atg9 between the TGN and endosomes. J Cell Sci. 2006;119:3888–900. 10.1242/jcs.03172. 16940348 10.1242/jcs.03172

[28] Mari M, Griffith J, Rieter E, Krishnappa L, Klionsky D, Reggiori F. An Atg9-containing compartment that functions in the early steps of autophagosome biogenesis. J Cell Biol. 2010;190:1005–22. 10.1083/jcb.200912089. 20855505 10.1083/jcb.200912089 PMC3101592

[29] van der Vaart A, Griffith J, Reggiori F. Exit from the Golgi Is Required for the Expansion of the Autophagosomal Phagophore in Yeast *Saccharomyces cerevisiae*. Mol Biol Cell. 2010;21:2270–84. 10.1091/mbc.E09-04-0345. 20444982 10.1091/mbc.E09-04-0345 PMC2893990

[30] Marcassa E, Raimondi M, Anwar T, Eskelinen E-L, Myers M, Triolo G, et al. Calpain mobilizes Atg9/Bif-1 vesicles from Golgi stacks upon autophagy induction by thapsigargin. Biol Open. 2017;6:551–62. 10.1242/bio.022806. 28302665 10.1242/bio.022806 PMC5450315

[31] Pavel M, Renna M, Park S, Menzies F, Ricketts T, Füllgrabe J, et al. Contact inhibition controls cell survival and proliferation via YAP/TAZ-autophagy axis. Nat commun. 2018;9:2961. 10.1038/s41467-018-05388-x. 30054475 10.1038/s41467-018-05388-x PMC6063886

[32] Mauthe M, Orhon I, Rocchi C, Zhou X, Luhr M, Hijkema K-J, et al. Chloroquine inhibits autophagic flux by decreasing autophagosome-lysosome fusion. Autophagy. 2018;14:1435–55. 10.1080/15548627.2018.1474314. 29940786 10.1080/15548627.2018.1474314 PMC6103682

[33] Tanida I, Ueno T, Kominami E. LC3 conjugation system in mammalian autophagy. Int J Biochem Cell Biol. 2004;36:2503–18. 10.1016/j.biocel.2004.05.009. 15325588 10.1016/j.biocel.2004.05.009 PMC7129593

[34] Pankiv S, Clausen T, Lamark T, Brech A, Bruun J-A, Outzen H, et al. p62/SQSTM1 binds directly to Atg8/LC3 to facilitate degradation of ubiquitinated protein aggregates by autophagy. J Biol Chem. 2007;282:24131–45. 10.1074/jbc.M702824200. 17580304 10.1074/jbc.M702824200

[35] Sahani M, Itakura E, Mizushima N. Expression of the autophagy substrate SQSTM1/p62 is restored during prolonged starvation depending on transcriptional upregulation and autophagy-derived amino acids. Autophagy. 2014;10:431–41. 10.4161/auto.27344. 24394643 10.4161/auto.27344 PMC4077882

[36] Hergovitch A. The Roles of NDR protein kinases in Hippo signalling. Genes. 2016;7:21. 10.3390/genes7050021. 27213455 10.3390/genes7050021 PMC4880841

[37] Yadav S, Puri S, Linstedt A. A primary role for golgi positioning in directed secretion, cell polarity, and wound healing. Mol Biol Cell. 2009;20:1728–36. 10.1091/mbc.E08-10-1077. 19158377 10.1091/mbc.E08-10-1077 PMC2655245

[38] Dubois F, Alpha K, Turner C. Paxillin regulates cell polarization and anterograde vesicle trafficking during cell migration. Mol Biol Cell. 2017;28:3815–31. 10.1091/mbc.E17-08-0488. 29046398 10.1091/mbc.E17-08-0488 PMC5739297

[39] Stegert M, Hergovich A, Tamaskovic R, Bichsel S, et Hemmings B. Regulation of NDR protein kinase by hydrophobic motif phosphorylation mediated by the mammalian Ste20-like kinase MST3. Mol Cell Biol. 2005;25:11019–29. 10.1128/MCB.25.24.11019-11029.2005. 16314523 10.1128/MCB.25.24.11019-11029.2005 PMC1316964

[40] Tang J, Ip J, Ye T, Ng Y-P, Yung W-H, Wu Z, et al. Cdk5-dependent Mst3 phosphorylation and activity regulate neuronal migration through RhoA inhibition. J Neurosci. 2014;34:7425–36. 10.1523/JNEUROSCI.5449-13.2014. 24872548 10.1523/JNEUROSCI.5449-13.2014 PMC6795244

[41] Mardakheh F, Self A, Marshall C. RHO bonding to FAM65A regulates Golgi reorientation during cell migration. J Cell Sci. 2016;129:4466–79. 10.1242/jcs.198614. 27807006 10.1242/jcs.198614 PMC5201024

[42] Guo W, Du K, Luo S, Hu D. Recent advances of autophagy in non-small cell lung cancer: from basic mechanisms to clinical application. Front Oncol. 2022;4:861959 10.3389/fonc.2022.861959. 10.3389/fonc.2022.861959 PMC9115384 35600411

[43] Dong S, Khoo A, Wei J, Bowser R, Weathington N, Xiao S, et al. Serum starvation regulates E-cadherin upregulation via activation of c-Src in non-small-cell lung cancer A549 cells. Am J Physiol Cell Physiol. 2014;307:C893–9. 10.1152/ajpcell.00132.2014. 25163517 10.1152/ajpcell.00132.2014 PMC4216942

[44] Joffre C, Codogno P, Fanto M, Hergovich A, Camonis J. STK38 at the crossroad between autophagy and apoptosis. Autophagy. 2016;12:594–5. 10.1080/15548627.2015.1135283. 26890257 10.1080/15548627.2015.1135283 PMC4835969

[45] Martin A, Jacquemyn M, Lipecka J, Chhuon C, Aushev V, Meunier B, et al. STK38 kinase acts as XPO1 gatekeeper regulating the nuclear export of autophagy proteins and other cargoes. EMBO Rep. 2019;20:e48150. 10.15252/embr.201948150. 31544310 10.15252/embr.201948150 PMC6832005

[46] Lu J, Feng Y, Li H, Li W, Chen H, Chen L. A review of nuclear Dbf2-related kinase 1 (NDR1) protein interaction as promising new target for cancer therapy. Int J Biol Macromol. 2024;259:129188 10.1016/j.ijbiomac.2023.129188. 38184050 10.1016/j.ijbiomac.2023.129188

[47] Jonischkies K, Del Angel M, Demiray YE, Zambrano A, Stork O. The NDR family of kinases: essential regulators of aging. Front Mol Neurosci. 2024;17:1371086 10.3389/fnmol.2024.1371086. 38803357 10.3389/fnmol.2024.1371086 PMC11129689

[48] Liu X-Y, Lu R, Chen J, Wang J, Qian H-M, Chen G, et al. Supressor of cytokine signaling 2 regulates retinal pigment epithelium metabolism by enhancing autophagy. Front Neurosci. 2021;15:738022. 10.3389/fnins.2021.738022. 34819832 10.3389/fnins.2021.738022 PMC8606588

[49] Paul I, Batth T, Iglesias-Gato D, Al-Araimi A, Al-Haddabi I, Alkharusi A, et al. The ubiquitin ligase Cullin5^SOCS2^ regulates NDR1/STK38 stability and NF-κB transactivation. Sci Rep. 2017;7:42800. 10.1038/srep42800. 28216640 10.1038/srep42800 PMC5316984

[50] Huang H, Han Q, Zheng H, Liu M, Shi S, Zhang T, et al. MAP4K4 mediates the SOX6-induced autophagy and reduces the chemosensitivity of cervical cancer. Cell Death Dis. 2021;13:13. 10.1038/s41419-021-04474-1. 34930918 10.1038/s41419-021-04474-1 PMC8688448

[51] Tang Y, Yu W. SIRT1 and p300/CBP regulate the reversible acetylation of serine-threonine kinase NDR2. Biochem Biophys Res Commun. 2019;518:396–401. 10.1016/j.bbrc.2019.08.069. 31427083 10.1016/j.bbrc.2019.08.069

[52] Patra S, Praharaj P, Singh A, Bhutia S. Targeting SIRT1-regulated autophagic cell death as a novel therapeutic avenue for cancer prevention. Drug Discov Today. 2023;28:103692 10.1016/j.drudis.2023.103692. 37379905 10.1016/j.drudis.2023.103692

[53] Hergovich A, Stegert M, Schmitz D, Vichalkovski A, Cornils H, Hemmings B. NDR kinases regulate essential cell processes from yeast to humans. Nat Rev Mol Cell Biol. 2009;7:253–64. 10.1038/nrm1891. 10.1038/nrm1891 16607288

[54] Schmitz-Rohmer D, Probst S, Yang Z-Z, Laurent F, Stadler M, Zuniga A, et al. NDR kinases are essential for somitogenesis and cardiac looping during mouse embryonic development. PLoS ONE. 2015;10:e0136566. 10.1371/journal.pone.0136566. 26305214 10.1371/journal.pone.0136566 PMC4549247

[55] Ma X, Wang D, Li N, Gao P, Zhang M, Zhang Y. Hippo kinase NDR2 inhibits IL-17 signaling by promoting Smurf1-mediated MEKK2 ubiquitination and degradation. Mol Immunol. 2019;105:131–6. 10.1016/j.molimm.2018.10.005. 30504095 10.1016/j.molimm.2018.10.005

[56] Hergovich A, Lamla S, Nigg E, Hemmings B. Centrosome-associated NDR kinase regulates centrosome duplication. Mol Cell. 2007;25:625–34. 10.1016/j.molcel.2007.01.020. 17317633 10.1016/j.molcel.2007.01.020

[57] Sagona A, Stenmark H. Cytokinesis and cancer. FEBS Lett. 2010;584:2652–61. 10.1016/j.febslet.2010.03.044. 20371245 10.1016/j.febslet.2010.03.044

[58] Wang L, Dynlacht B. The regulation of cilium assembly and disassembly in development and disease. Development. 2018;145:dev151407. 10.1242/dev.151407. 30224385 10.1242/dev.151407 PMC6176931

[59] Chiba S, Amagai Y, Homma Y, Fukuda M, Mizuno K. NDR2-mediated Rabin8 phosphorylation is crucial for ciliogenesis by switching binding specificity from phosphatidylserine to Sec15. EMBO J. 2013;32:874–85. 10.1038/emboj.2013.32. 23435566 10.1038/emboj.2013.32 PMC3604723

[60] Rehberg K, Kliche S, Madencioglu D, Thiere M, Müller B, Meineke B, et al. The Serine/Threonine Kinase Ndr2 Controls Integrin Trafficking and Integrin-Dependent Neurite Growth. J Neurosci. 2014;34:5342–54. 10.1523/JNEUROSCI.2728-13.2014. 24719112 10.1523/JNEUROSCI.2728-13.2014 PMC6609001

[61] Demiray Y, Rehberg K, Kliche S, Stork O. Ndr2 kinase controls neurite outgrowth and dendritic branching through α1 integrin expression. Front Mol Neurosci. 2018;11:66. 10.3389/fnmol.2018.00066. 29559888 10.3389/fnmol.2018.00066 PMC5845635

[62] Claude-Taupin A, Isnard P, Bagattin A, Kuperwasser N, Roccio F, Ruscica B, et al. The AMPK-Sirtuin 1-YAP axis is regulated by fluid flow intensity and controls autophagy flux in kidney epithelial cells. Nat Commun. 2023;14:8056. 10.1038/s41467-023-43775-1. 38052799 10.1038/s41467-023-43775-1 PMC10698145

[63] Sun T, Li D, Wang L, Xia L, Ma J, Feng G, et al. c-Jun NH2-terminal kinase activation is essential for up-regulation of LC3 during ceramide-induced autophagy in human nasopharyngeal carcinoma cells. J Transl Med. 2011;26:161. 10.1186/1479-5876-9-161. 10.1186/1479-5876-9-161 PMC3189397 21943220

[64] Gosavi P, Houghton F, McMillan P, Hanssen E, Gleeson P. The Golgi ribbon in mammalian cells negatively regulates autophagy by modulating mTOR activity. J Cell Sci. 2018;131:jcs211987. 10.1242/jcs.211987. 29361552 10.1242/jcs.211987

[65] Chang H-Y, Yang WY. Golgi quality control and autophagy. IUBMB Life. 2022;74:361–70. 10.1002/iub.2611. 35274438 10.1002/iub.2611

[66] Lamb C, Yoshimori T, Tooze S. The autophagosome: origins unknown, biogenesis complex. Nat Rev Mol Cell Biol. 2013;14:759–74. 10.1038/nrm3696. 24201109 10.1038/nrm3696

[67] Choi J, Jang H, Xuan Z, Park D. Emerging roles of ATG9/ATG9A in autophagy: implications for cell and neurobiology. Autophagy. 2024;20:2373–87. 10.1080/15548627.2024.2384349. 39099167 10.1080/15548627.2024.2384349 PMC11572220

[68] Binotti B, Ninov M, Cepeda AP, Ganzella M, Matti U, Riedel D, et al. ATG9 resides on a unique population of smal vesicles in presynaptic nerve terminals. Autophagy. 2023;20:883–901. 10.1080/15548627.2023.2274204. 37881948 10.1080/15548627.2023.2274204 PMC11062364

[69] Sharifi MN, Mowers EE, Drake LE, Collier C, Chen H, Zamora M, et al. Autophagy Promotes Focal Adhesion Disassembly and Cell Motility of Metastatic Tumor Cells through the Direct Interaction of Paxillin with LC3. Cell Rep. 2016;15:1660–72. 10.1016/j.celrep.2016.04.065. 27184837 10.1016/j.celrep.2016.04.065 PMC4880529

[70] Kang R, Zeh HJ, Lotze MT, Tang D. The Beclin 1 network regulates autophagy and apoptosis. Cell Death Differ. 2011;18:571–80. 10.1038/cdd.2010.191. 21311563 10.1038/cdd.2010.191 PMC3131912

[71] Deng H, Deng L, Chao H, Yu Z, Huang J, Song Z, et al. RAB14 promotes epithelial-mesenchymal transition in bladder cancer through autophagy‑dependent AKT signaling pathway. Cell Death Discov. 2023;9:292. 10.1038/s41420-023-01579-8. 37558664 10.1038/s41420-023-01579-8 PMC10412633

[72] Alizadeh J, Glogowska A, Thliveris J, Kalantari F, Shojaei S, Hombach-Klonisch S, et al. Autophagy modulates transforming growth factor beta 1 induced epithelial to mesenchymal transition in non-small cell lung cancer cells. Biochim Biophys Acta Mol Cell Res. 2018;1865:749–68. 10.1016/j.bbamcr.2018.02.007. 29481833 10.1016/j.bbamcr.2018.02.007

[73] Millarte V, Farhan H. The Golgi in cell migration: regulation by signal transduction and its implications for cancer cell metastasis. ScientificWorldJournal. 2012;2012:498278. 10.1100/2012/498278. 22623902 10.1100/2012/498278 PMC3353474

[74] Bui S, Mejia I, Diaz B, et Wang Y. Adaptation of the Golgi apparatus in cancer cell invasion and metastasis. Front Cell Dev Biol. 2021;10:806482. 10.3389/fcell.2021.806482. 10.3389/fcell.2021.806482 PMC8703019 34957124

[75] Darido C, Jane S. Golgi feels its own wound. Adv Wound Care. 2013;2:87–92. 10.1089/wound.2011.0352. 10.1089/wound.2011.0352 PMC3840545 24527331

[76] Campisi D, Desrues L, Dembélé K-P, Mutel A, Parment R, Gandolfo P, et al. The core autophagy protein ATG9A controls dynamics of cell protrusions and directed migration. J Cell Biol. 2022;221:e202106014. 10.1083/jcb.202106014. 35180289 10.1083/jcb.202106014 PMC8932524

