# Supplementary material for: NDR2 regulates non-small cell lung cancer cell migration under starvation by supporting autophagosome biogenesis through LC3 and ATG9A regulation
Source: Cell Death Discov. 2025 Dec 13;12:50. doi: 10.1038/s41420-025-02889-9 (PMC12847810; doi:10.1038/s41420-025-02889-9)
Supplement: Supplementary file 1 — Figues S1_S8 [file 41420_2025_2889_MOESM1_ESM.pdf]

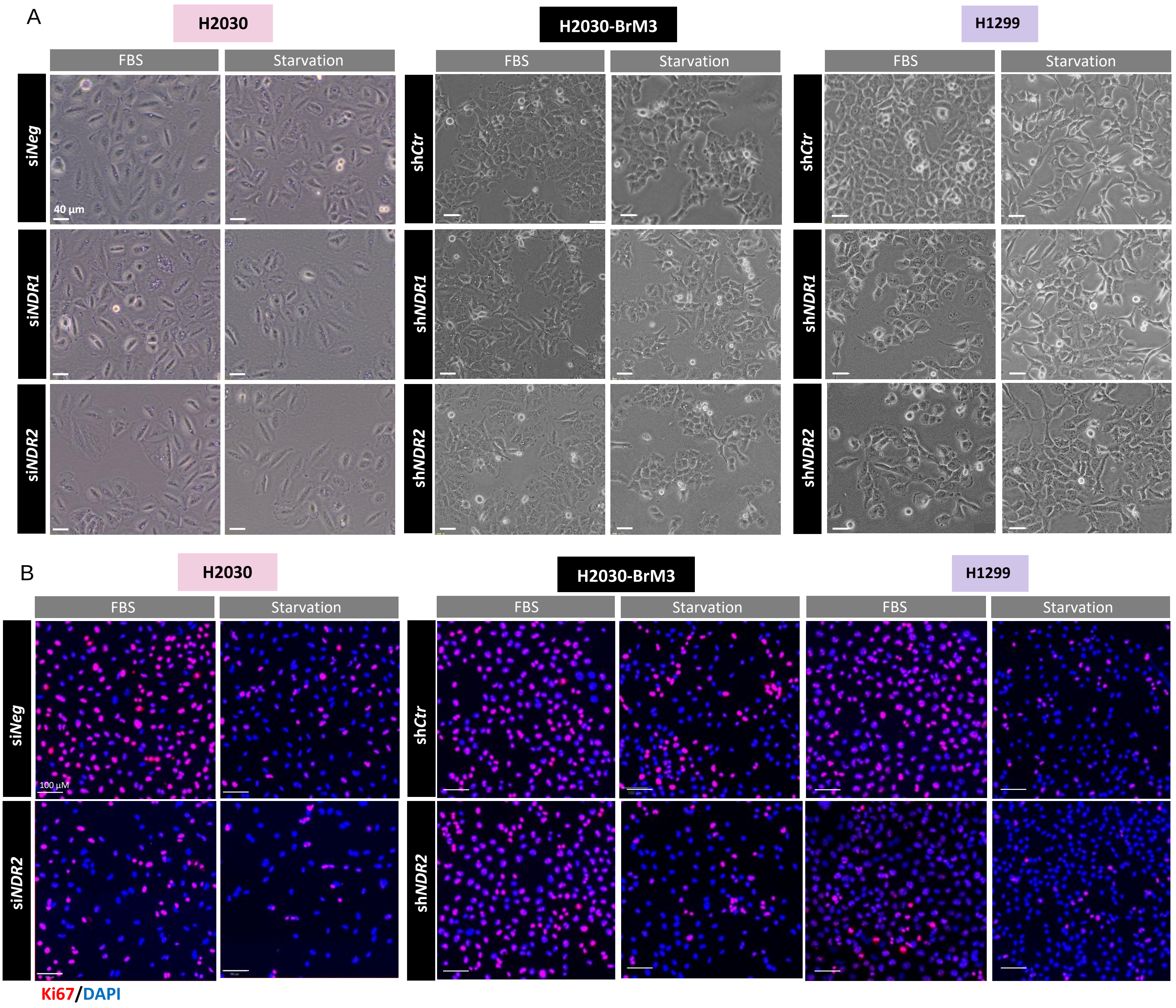

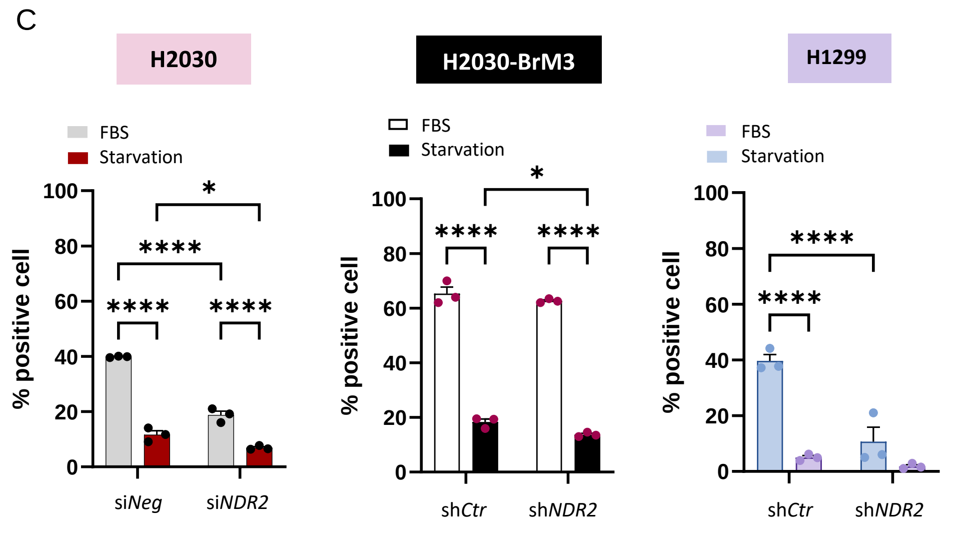

Figure S1. The loss of NDR2 induced a decrease in proliferation under serum deprivation and normal conditions.

(**A**) Phase contrast images of the H2030, H2030-BrM3 and H1299 cell lines following serum deprivation and/or the depletion of NDR1 or NDR2. (**B**) Labeling of H2030 cells with the proliferation marker Ki67 and (**C**) quantification of Ki67 levels in H2030, H2030-BrM3 and H1299 cells. Two-way ANOVA followed by Tukey's post hoc test were used to analyze the data, means ± SEMs, N=3, *p<0.05, **p<0.01, ***p<0.001 and ****p<0.0001.

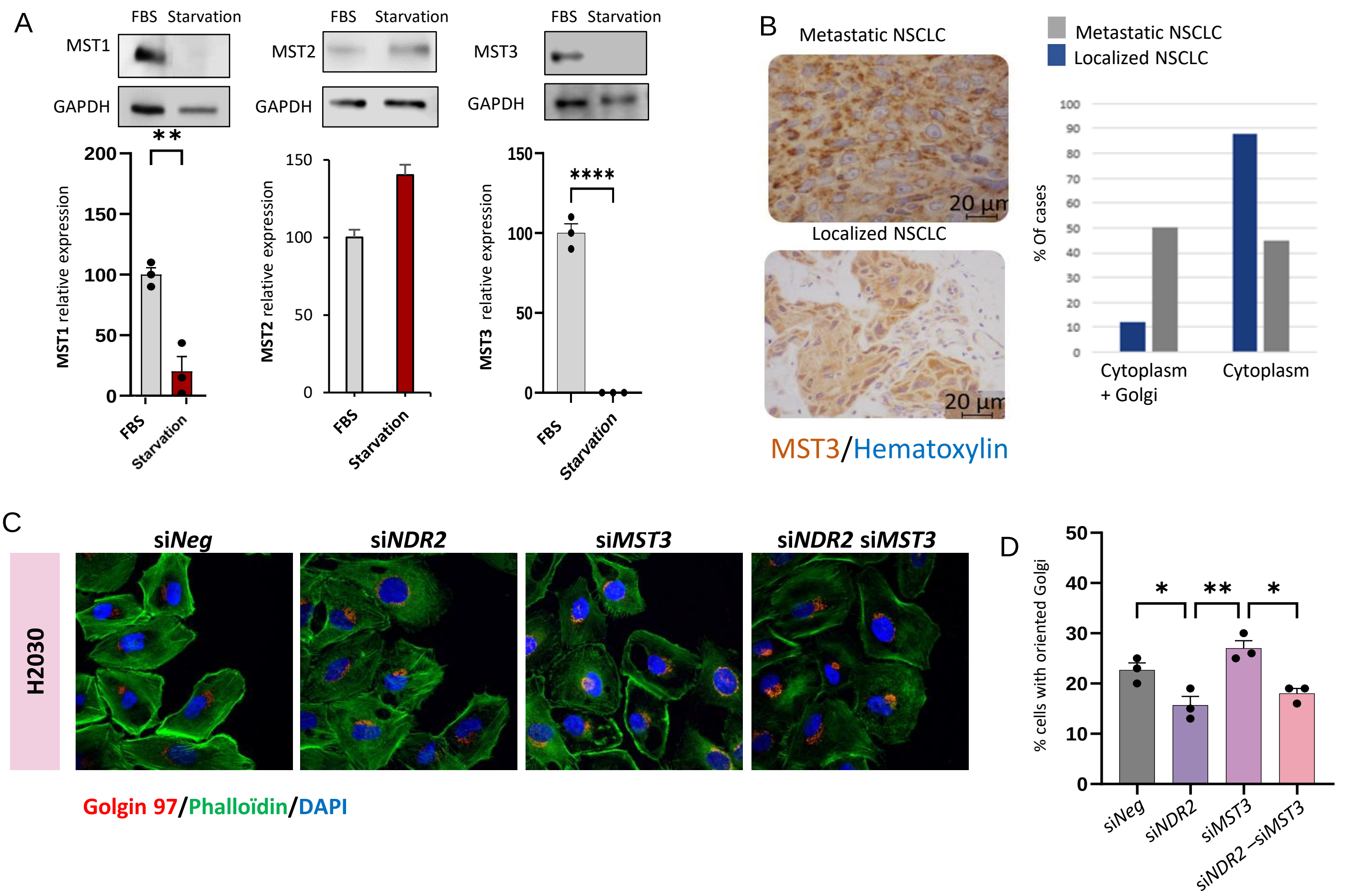

Figure S2. MST kinases are not involved in regulating NDR2 activity during serum deprivation.

(**A**) Western blots and quantification of MST1, MST2 and MST3 expression following 24 h of serum deprivation or normal cultivation. (**B**) MST3 labeling in metastatic or nonmetastatic NSCLC samples and quantification of MST3 expression in the Golgi or cytoplasm. (**C**) Image showing the position of the Golgi (red) during migration (t = 6 h) and (**D**) quantification of cells with the Golgi oriented toward the migration front during serum deprivation with or without the loss of NDR2 or MST3. Two-way ANOVA followed by Tukey's post hoc test were used to analyze the data, means ± SEMs, N=3, *p<0.05, **p<0.01, ***p<0.001 and ****p<0.0001.

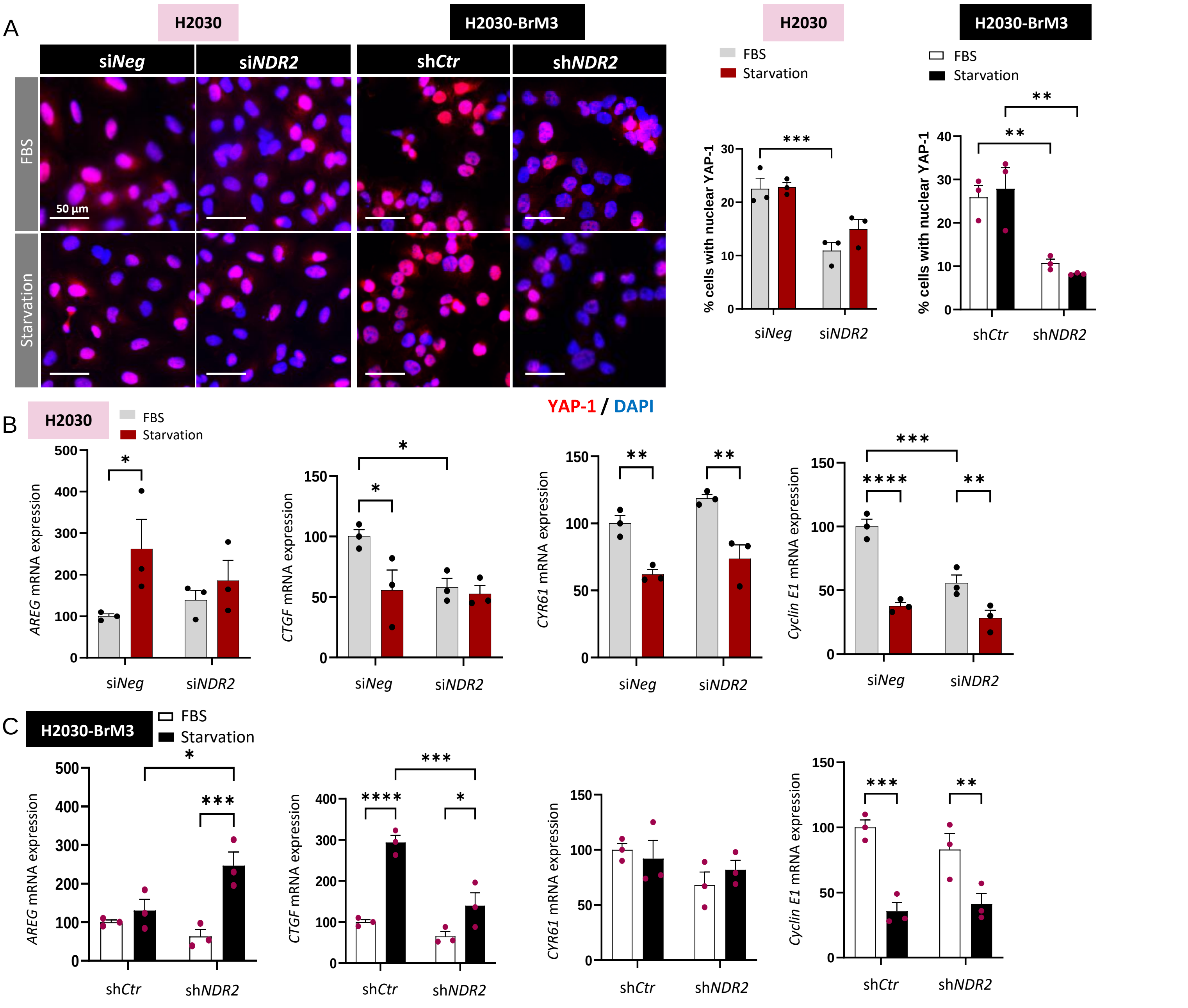

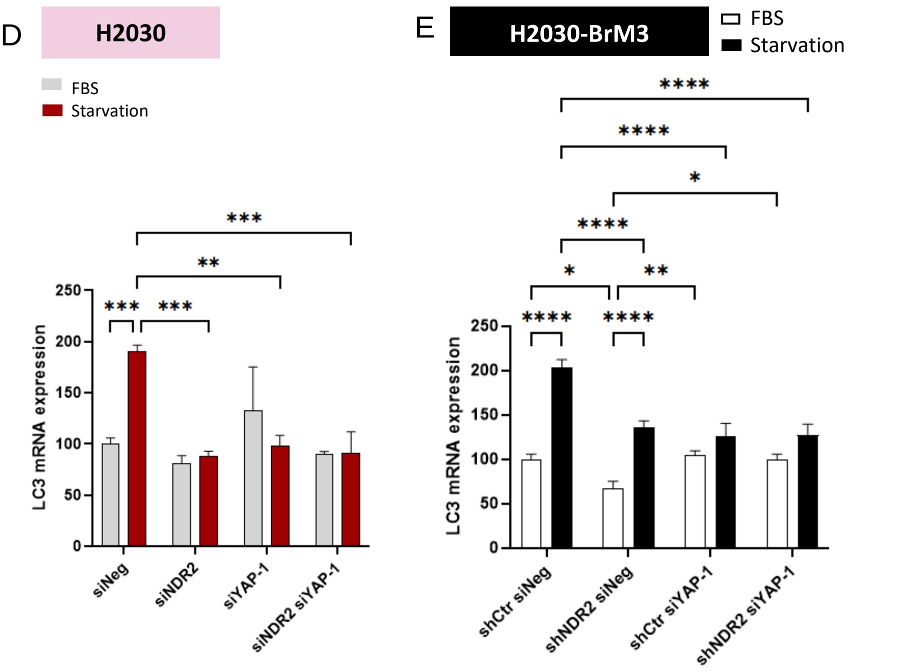

Figure S3. The loss of NDR2 affected YAP-1 activity under starvation conditions.

NDR2-silenced (si*NDR2* or sh*NDR2*) or control (si*Neg* or sh*Ctr*) or YAP-1-silenced (si*YAP-1*) H2030 and H2030-BrM3 cells were subjected to 24 h of FBS deprivation or normal culture. (**A**) YAP-1 labeling and quantification of nuclear labeling. (**B-C**) Expression of various YAP-1 target genes in H2030 cells (**B**) and H2030-BrM3 cells (**C**). LC3 mRNA expression in H2030 cells (**D**) and H2030-BrM3 cells (**E**). Two-way ANOVA followed by Tukey's post hoc test were used to analyze the data, means ± SEMs, N=3, *p<0.05, **p<0.01, and ***p<0.001

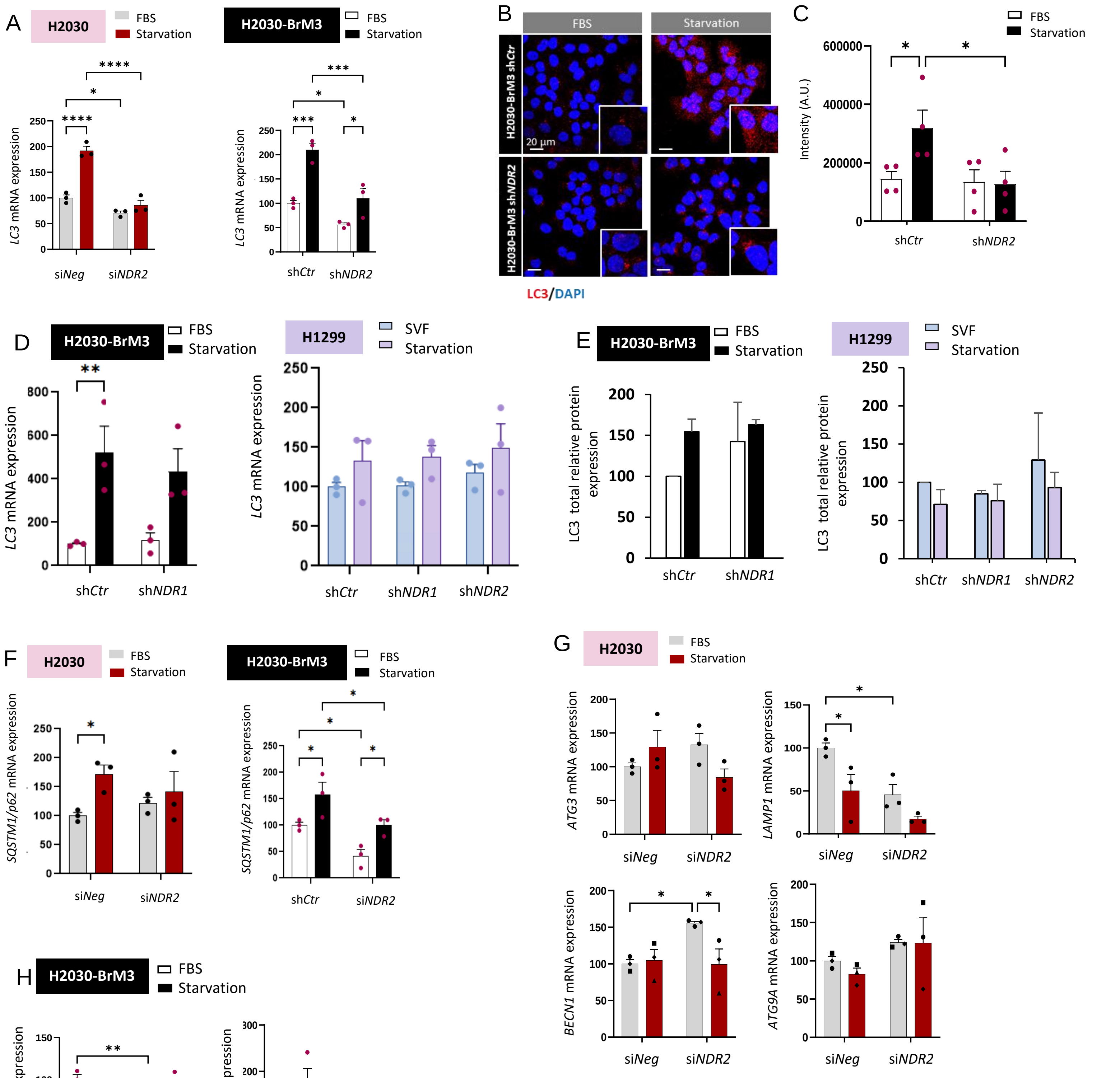

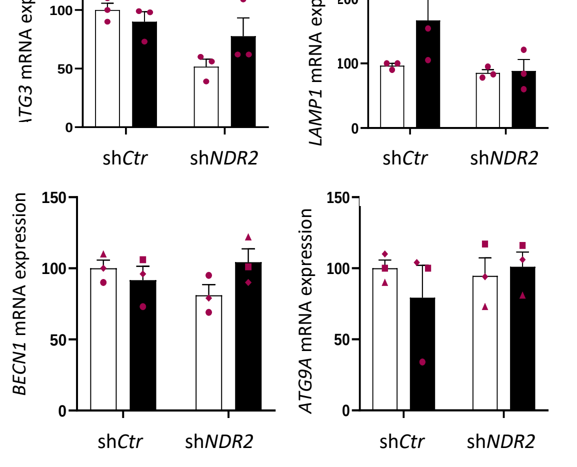

Figure S4. NDR2 had no impact on TFEB activity.

(**A**) Quantification of *LC3* mRNA expression in H2030 and H2030-BrM3 lines expressing or lacking NDR2 following 24 h of serum deprivation. (**B**) Image of LC3 immunofluorescence labeling of H2030-BrM3 cells in which NDR2 was or was not depleted with an shRNA under serum starvation or complete serum conditions and quantification of the results (**C**). (**D**) *LC3* mRNA expression in H2030-BrM3 and H1299 cells cultured with or without FBS (starvation) and depleted of NDR2 or NDR1. (**E**) LC3 protein expression in H2030-BrM3 and H1299 cells cultured with or without FBS and depleted of NDR2 or NDR1. (**F**) Quantification of *SQSTM1/p62* mRNA expression in H2030 and H2030-BrM3 cells. ((**G-H**) Expression of different TFEB target genes in H2030 (**G**) and H2030-BrM3 cells (**H**). Two-way ANOVA followed by Tukey's post hoc test were used to analyze the data, means ± SEMs, N=3, *p<0.05 and **p<0.01

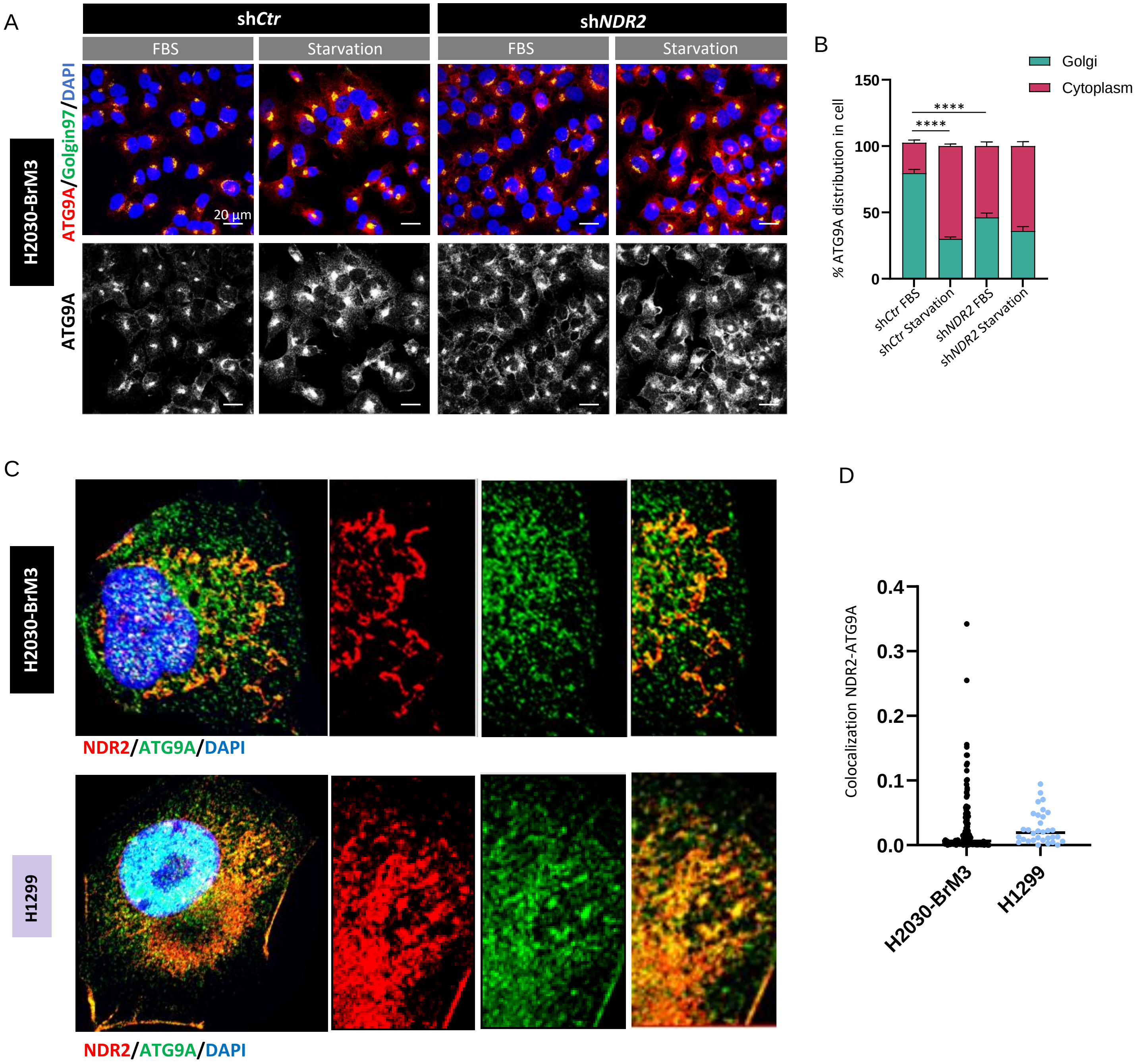

Figure S5. NDR2 interacted with ATG9A for transport into H2030-BrM3 and H1299 cells. Immunolabelling (**A**) and quantification of ATG9A localization (**B**) following 24 h or culture with or without FBS along with NDR2 silencing in H2030 cells. (**C**) Colocalization of NDR2 and ATG9A immunolabeling in H2030 cells under normal or serum-deprived conditions and after the transfection of si*NDR2* or si*ATG9A.* (**D**) Quantification of NDR2-ATG9A colocalization.

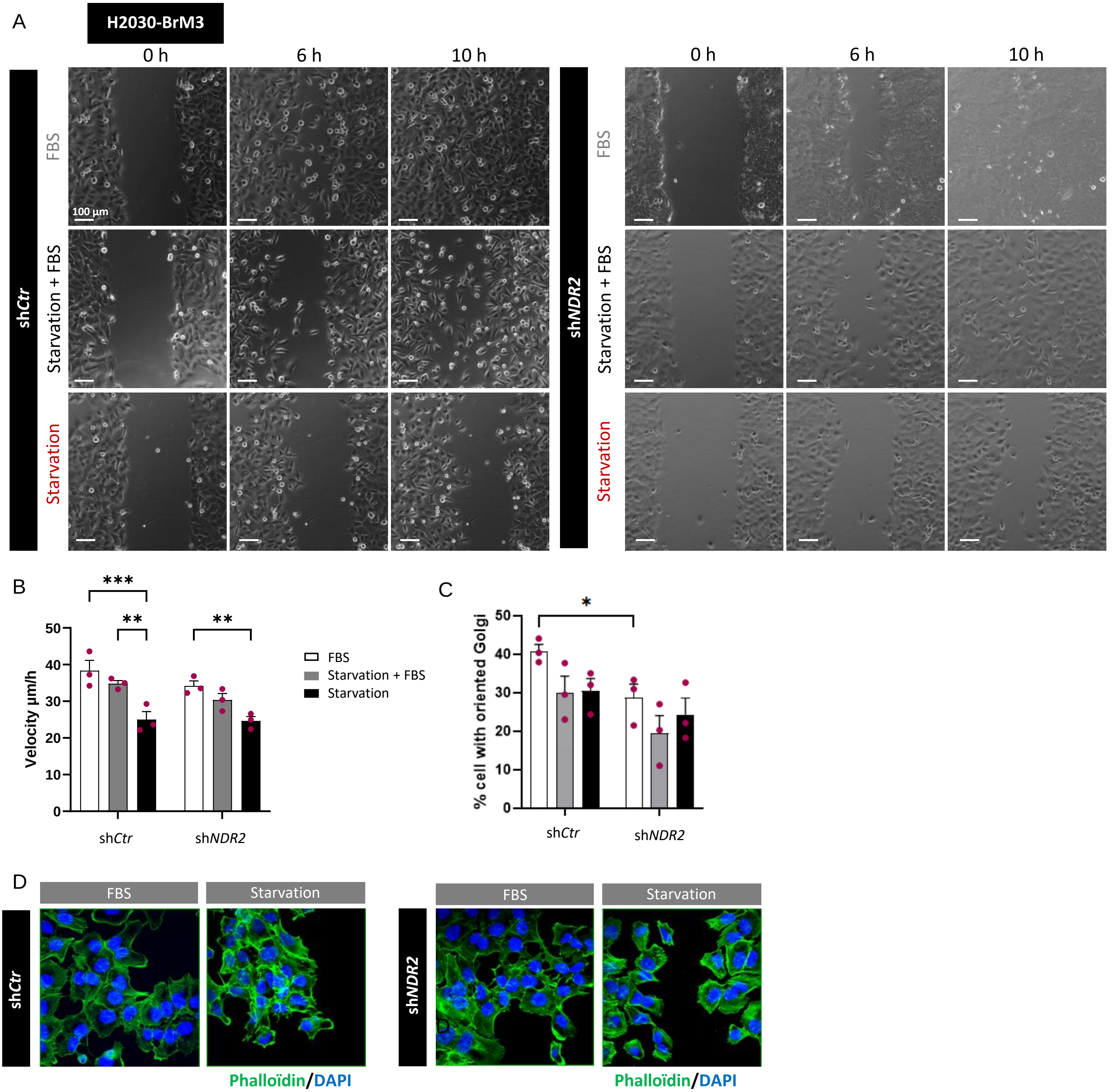

Figure S6. NDR2 is required for H2030-BrM3 cell migration under serum starvation conditions.

(**A**) Inverted phase microscopy images (x20) and (**B**) quantification of the results of the wound-healing assay with H2030-BrM3 cells with or without NDR2 silencing in complete media or starvation media at 0 h, 6 h and 10 h after scraping. (**C**) Quantification of the number of cells with or without NDR2 silencing in which the Golgi was oriented toward the migration front during serum deprivation. (**D**) Immunolabeling for phalloidin (green). Two-way ANOVA followed by Tukey's post hoc test were used to analyze the data, means ± SEMs, N=3, *p<0.05 and **p<0.01.

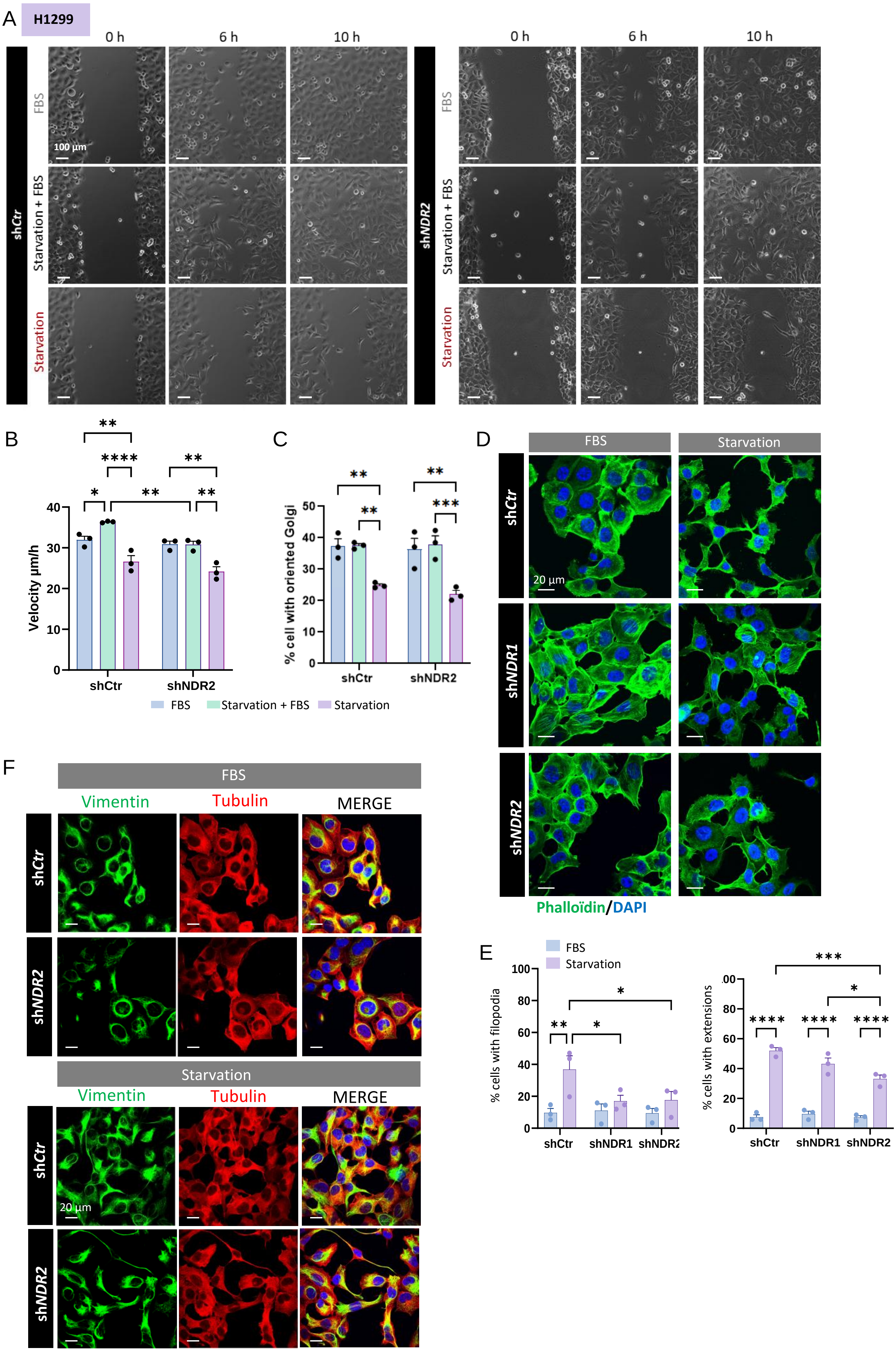
A
H1299
0 h
6 h
10 h
shCtr
FBS
Starvation + FBS
Starvation
100 µm
shNDR2
B
Velocity µm/h
shCtr
shNDR2
C
% cell with oriented Golgi
FBS
Starvation + FBS
Starvation
D
shCtr
shNDR1
shNDR2
20 µm
Phalloïdin/DAPI
E
% cells with filopodia
% cells with extensions
F
Vimentin
Tubulin
MERGE

Figure S7. NDR2 is required for H1299 cell migration and regulates the cytoskeleton under serum starvation conditions.

(**A**) Inverted phase contrast microscopy (x20) images and (**B**) quantification of the wound-healing assay with H1299 cells with or without NDR2 and/or ATG9A silencing cultured in complete media or starvation media at 0 h, 6 h and 10 h after scraping. (**C**) Quantification of the number of cells with NDR2 silencing in which the Golgi was oriented toward the migration front during serum deprivation. (**D**) Immunolabeling for phalloidin (green) and quantification of cell expansion and filopodia (**E**). (**F**) Immunolabeling for vimentin (green) and tubulin (red). Two-way ANOVA followed by Tukey's post hoc test were used to determine the means ± SEMs, N=3. *$p<0.05$, **$p<0.01$, ***$p<0.001$ and ****$p<0.0001$.

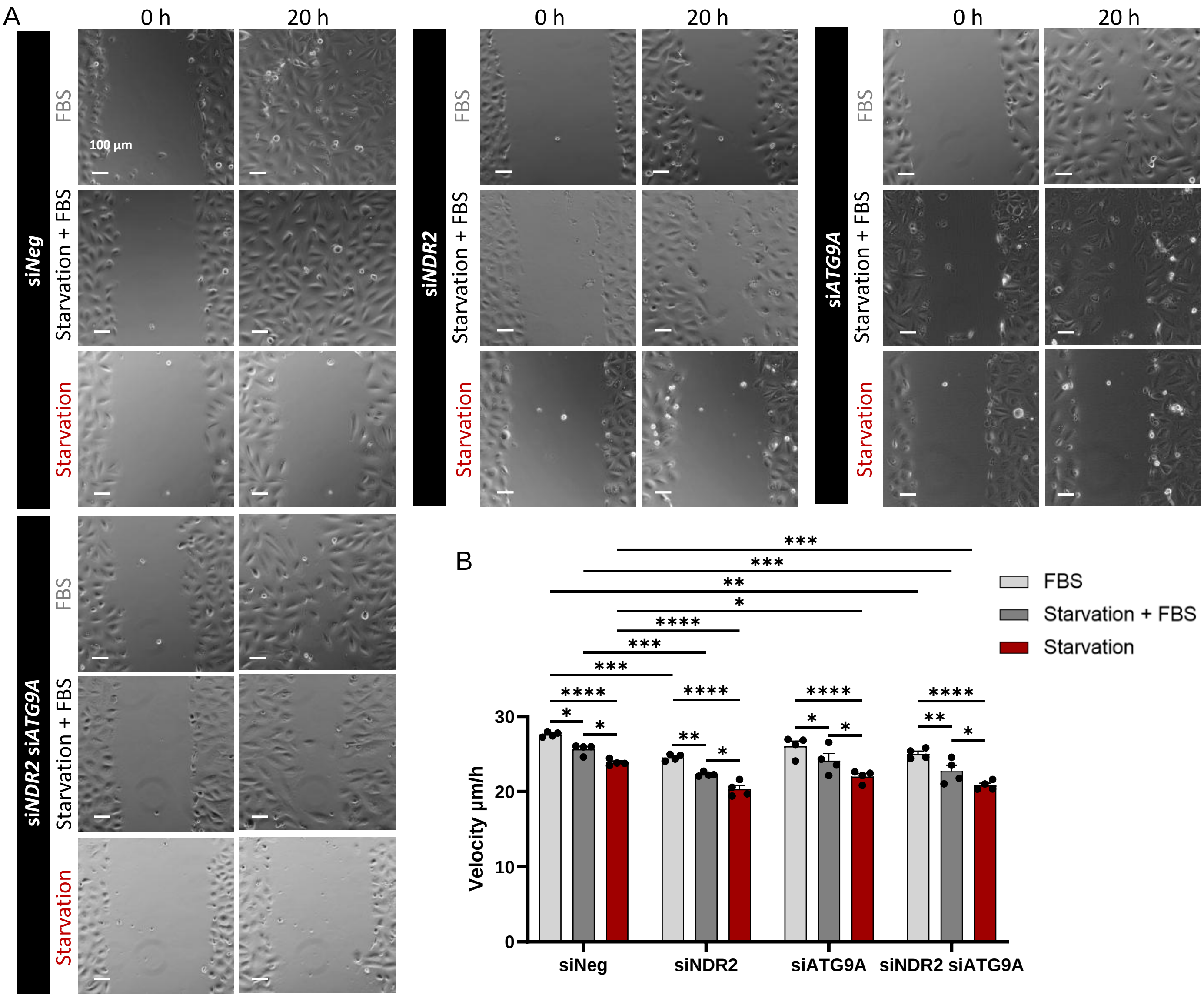

Figure S8. NDR2 and ATG9A are required for H1299 cell migration
(**A**) Inverted phase microscopy images (x20) and (**B**) quantification of the wound-healing assay with H2030 cells with or without NDR2 and/or ATG9A silencing in full media or starvation media and with or without CQ at 0 h, 10 h and 20 h after scraping. Two-way ANOVA followed by Tukey's post hoc test were used to determine the means ± SEMs, N=4. *p<0.05, **p<0.01, ***p<0.001 and ****p<0.0001.
