# Supplementary material for: NDR2 regulates non-small cell lung cancer cell migration under starvation by supporting autophagosome biogenesis through LC3 and ATG9A regulation
Source: Cell Death Discov. 2025 Dec 13;12:50. doi: 10.1038/s41420-025-02889-9 (PMC12847810; doi:10.1038/s41420-025-02889-9)
Supplement: Supplementary file 2 — Tables S1_S3 [file 41420_2025_2889_MOESM2_ESM.pdf]

Table S1. siRNA and plasmids used in this work.

Target	siRNA (5' → 3')
**NDR1**	**si1:** AAGUAAUAGGCAGAGGAGCAU(TT)<br>**si2:** AAGAGCAGGUUGGCCACAUUC(TT)
**NDR2**	**si1:** AAGUUACGUCGAUCACAACAC(TT)<br>**si2:** AAGACACCUUGACAGAAGAGG (TT)
**ATG9A**	**si1:**UUCUGCGUCUGCACGAUCC(TT)<br>**si2:** UUUCGGAAGAAGUCUAUA(TT)
**YAP-1**	**si1**: AGGUACUUCCUCAAUCACA (TT)<br>**si2**: CUAGGAAGGCGAUGAAUCA (TT)
**MST3**	**si1:** GGCAUUGACAAUCGGACUC(TT)<br>**si2:** GGAGAAGAGCCAGGCGUGC(TT)

Table S2. Primers used for PCR and qRT-PCR in this work.

Target	Primers (5' → 3 ')
**ATG3**	**F:** ACT-GAT-GCT-GGC-GGT-GAA-GAT-G<br>**R:** GTG-CTC-AAC-TGT-TAA-AGG-CTG-CC
**ATG9A**	**F:** GAGCCTGCATGCCCTCTATA<br>**R:** CAGAGCGAGGGATAGACTGG
**Beclin-1**	**F:** GGCTGAGAGACTGGATCAGG<br>**R:** CTGCGTCTGGGCATAACG
**LAMP1**	**F:** CGT-GTC-ACG-AAG-GCG-TTT-TCA-G<br>**R:** CTG-TTC-TCG-TCC-AGC-AGA-CAC-T
**LC3B**	**F:** GAGAAGCAGCTTCCTGTTCTGG<br>**R:** GTGTCCGTTCACCAACAGGAAC
**NDR1**	**F:** CTGGAGCCAGTTCTGCTTCT<br>**R:** GTCACGCTCCGCACGAAT
**NDR2**	**F:** CTTGGCTTGGATGACTTTGAG<br>**R:** AGGCACCATCTGCTTCTACC
**SQSTM1/p62**	**F**: GTG-CTC-AAC-TGT-TAA-AGG-CTG-CC<br>**R**: AGT-GTC-CGT-GTT-TCA-CCT-TCC-G
**S16**	**F:** CTG GAG CCA GTT CTG CTT CT<br>**R:** TCT GGT AAT AGG CCA CCA GG

Table S3. Antibodies used in this work.

Target, clone	Host	Clonality	Supplier, product number
**ATG9A**	Rabbit	Monoclonal	Abcam, Ab-108338
**E-Cadherin (24E10)**	Rabbit	Monoclonal	Cell Signaling Technology, 3195S
**Golgin-97**	Mouse		Santa Cruz Biotechnology, SC-73619
**GAPDH**	Rabbit	Monoclonal	Cell Signaling Technology, 2118S
**Ki67 (8D5)**	Mouse	Monoclonal	Cell Signaling Technology, 9449S
**LC3B**	Rabbit	Monoclonal	Cell Signaling Technology, 3868S
**MST3**	Rabbit	Polyclonal	Cell Signaling Technology, 3723S
**N-Cadherin**	Mouse	Monoclonal	Ebioscience, 14-3259-82
**NDR1 (YJ-7)**	Mouse	Monoclonal	Santa Cruz Biotechnology FO316, sc-100404
**NDR2 (STK38L)**	Mouse	Monoclonal	Lifespan Biosciences, LS-C174201-100
**Phalloidin**			Invitrogen, Thermofisher scientific, A12379
**SQSTM1/p62**	Rabbit	Polyclonal	Sigma Aldrich, P0067
**Tubulin**	Mouse	Monoclonal	Sigma Aldrich, 038M4837V
**SQSTM1/p62**	Rabbit		Sigma Aldrich, P0067
**Vimentin**	Rabbit	Monoclonal	Cell Signaling Technology, 5741S
**YAP(D8H1X)**	Rabbit	Monoclonal	Cell Signaling Technology, 14074S
**Anti-Mouse IgG HRP linked antibody**	Goat		Cell Signaling Technology, 7076S
**Anti-Rabbit IgG HRP linked antibody**	Goat		Cell Signaling Technology, 7074S
