# Supplementary material for: NDR2 regulates non-small cell lung cancer cell migration under starvation by supporting autophagosome biogenesis through LC3 and ATG9A regulation
Source: Cell Death Discov. 2025 Dec 13;12:50. doi: 10.1038/s41420-025-02889-9 (PMC12847810; doi:10.1038/s41420-025-02889-9)
Supplement: Supplementary file 3 — Original Data [file 41420_2025_2889_MOESM3_ESM.pdf]

Figure 1C/D

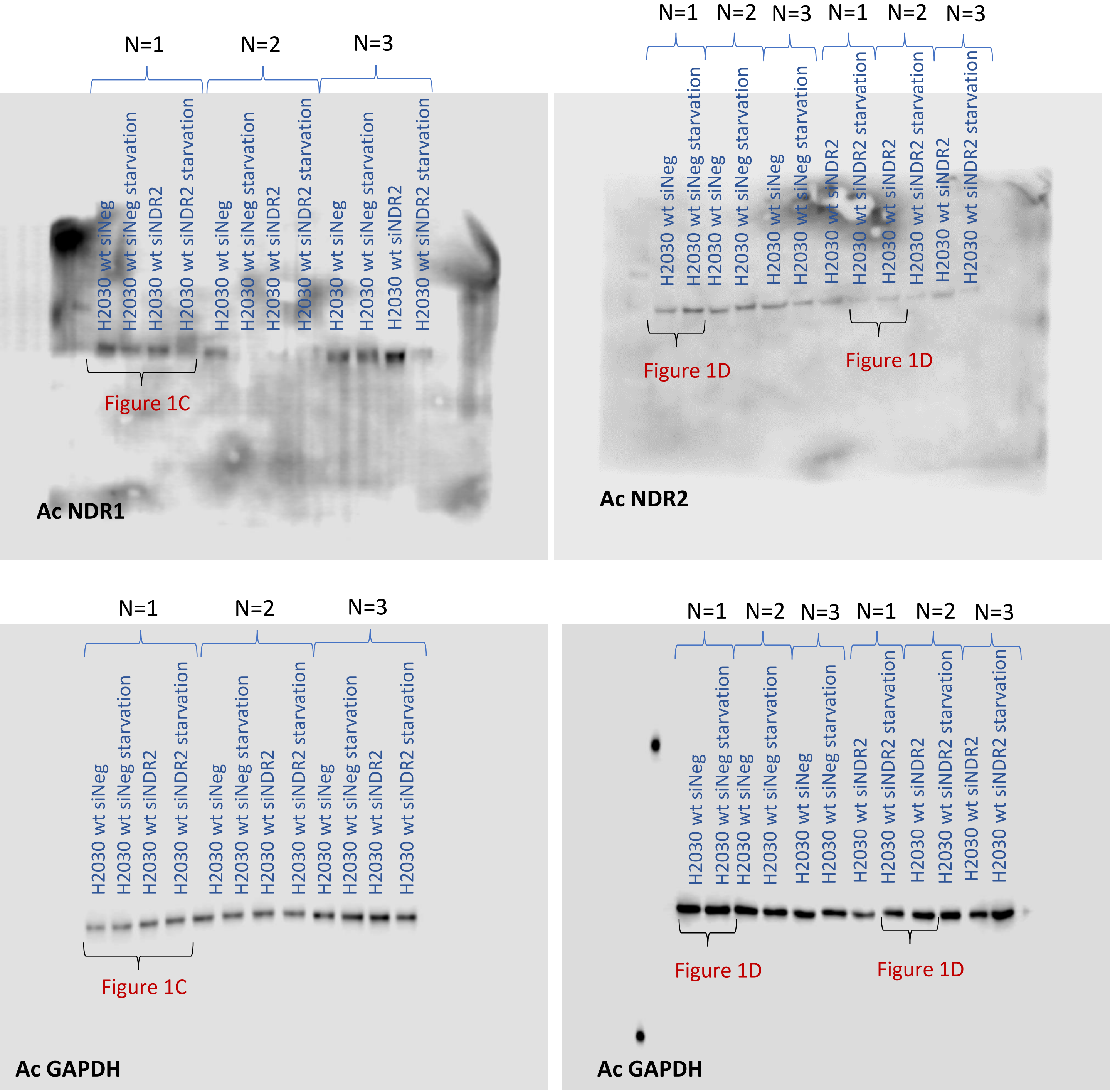
N=1
N=2
N=3
H2030 wt siNeg
H2030 wt siNeg starvation
H2030 wt siNDR2
H2030 wt siNDR2 starvation
H2030 wt siNeg
H2030 wt siNeg starvation
H2030 wt siNDR2
H2030 wt siNDR2 starvation
H2030 wt siNeg
H2030 wt siNeg starvation
H2030 wt siNDR2
H2030 wt siNDR2 starvation
Figure 1C
Ac NDR1
N=1
N=2
N=3
N=1
N=2
N=3
H2030 wt siNeg
H2030 wt siNeg starvation
H2030 wt siNeg
H2030 wt siNeg starvation
H2030 wt siNeg
H2030 wt siNeg starvation
H2030 wt siNDR2
H2030 wt siNDR2 starvation
H2030 wt siNDR2
H2030 wt siNDR2 starvation
H2030 wt siNDR2
H2030 wt siNDR2 starvation
Figure 1D
Figure 1D
Ac NDR2
N=1
N=2
N=3
H2030 wt siNeg
H2030 wt siNeg starvation
H2030 wt siNDR2
H2030 wt siNDR2 starvation
H2030 wt siNeg
H2030 wt siNeg starvation
H2030 wt siNDR2
H2030 wt siNDR2 starvation
H2030 wt siNeg
H2030 wt siNeg starvation
H2030 wt siNDR2
H2030 wt siNDR2 starvation
Figure 1C
Ac GAPDH
N=1
N=2
N=3
N=1
N=2
N=3
H2030 wt siNeg
H2030 wt siNeg starvation
H2030 wt siNeg
H2030 wt siNeg starvation
H2030 wt siNeg
H2030 wt siNeg starvation
H2030 wt siNDR2
H2030 wt siNDR2 starvation
H2030 wt siNDR2
H2030 wt siNDR2 starvation
H2030 wt siNDR2
H2030 wt siNDR2 starvation
Figure 1D
Figure 1D
Ac GAPDH

Figure 1C/D

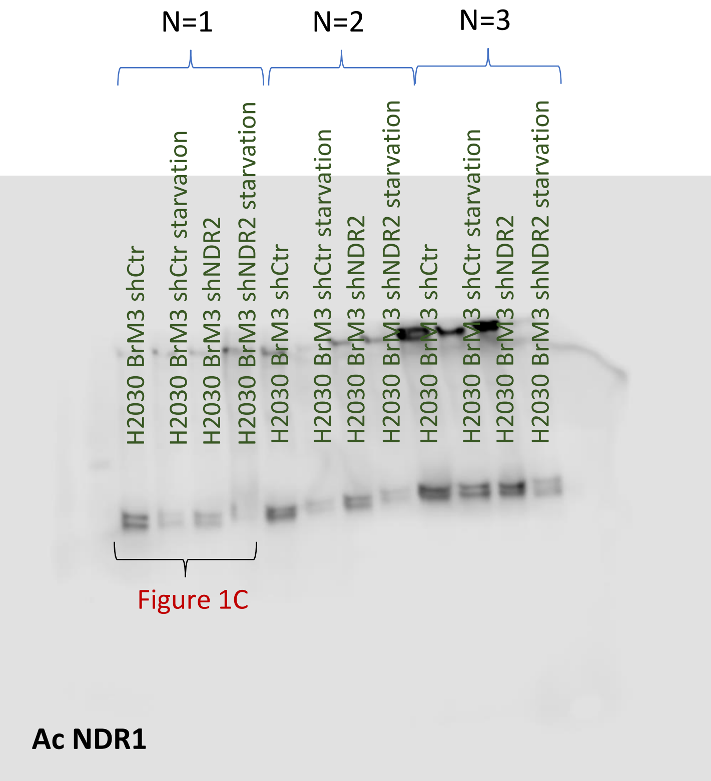

N=1
N=2
N=3
H2030 BrM3 shCtr
H2030 BrM3 shCtr starvation
H2030 BrM3 shNDR2
H2030 BrM3 shNDR2 starvation
H2030 BrM3 shCtr
H2030 BrM3 shCtr starvation
H2030 BrM3 shNDR2
H2030 BrM3 shNDR2 starvation
H2030 BrM3 shCtr
H2030 BrM3 shCtr starvation
H2030 BrM3 shNDR2
H2030 BrM3 shNDR2 starvation
Figure 1C
Ac NDR1

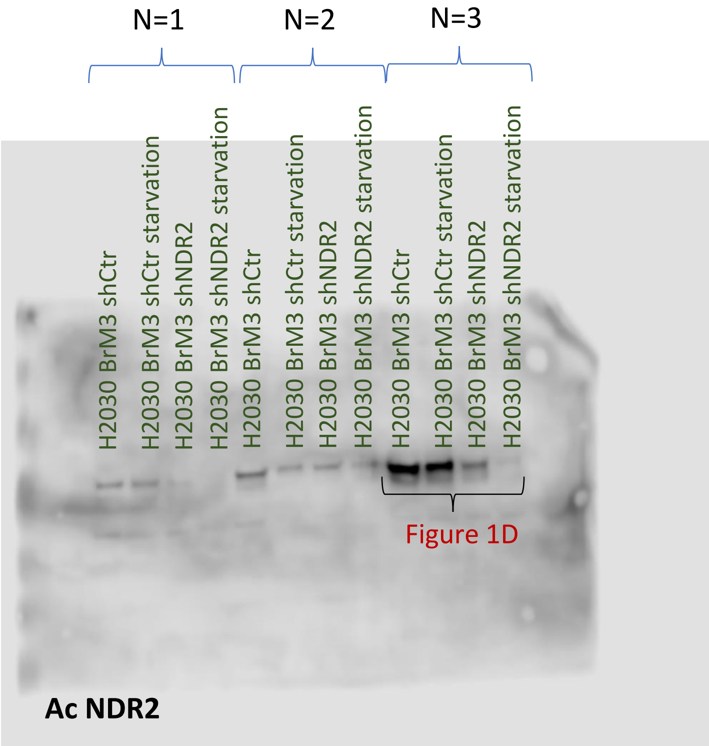

N=1
N=2
N=3
H2030 BrM3 shCtr
H2030 BrM3 shCtr starvation
H2030 BrM3 shNDR2
H2030 BrM3 shNDR2 starvation
H2030 BrM3 shCtr
H2030 BrM3 shCtr starvation
H2030 BrM3 shNDR2
H2030 BrM3 shNDR2 starvation
H2030 BrM3 shCtr
H2030 BrM3 shCtr starvation
H2030 BrM3 shNDR2
H2030 BrM3 shNDR2 starvation
Figure 1D
Ac NDR2

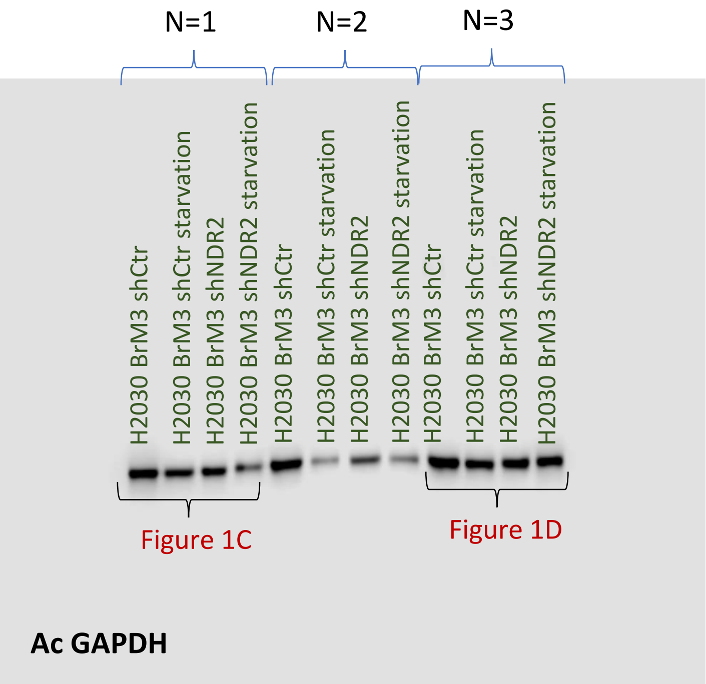

N=1
N=2
N=3
H2030 BrM3 shCtr
H2030 BrM3 shCtr starvation
H2030 BrM3 shNDR2
H2030 BrM3 shNDR2 starvation
H2030 BrM3 shCtr
H2030 BrM3 shCtr starvation
H2030 BrM3 shNDR2
H2030 BrM3 shNDR2 starvation
H2030 BrM3 shCtr
H2030 BrM3 shCtr starvation
H2030 BrM3 shNDR2
H2030 BrM3 shNDR2 starvation
Figure 1C
Figure 1D
Ac GAPDH

Figure 1C/D

H1299

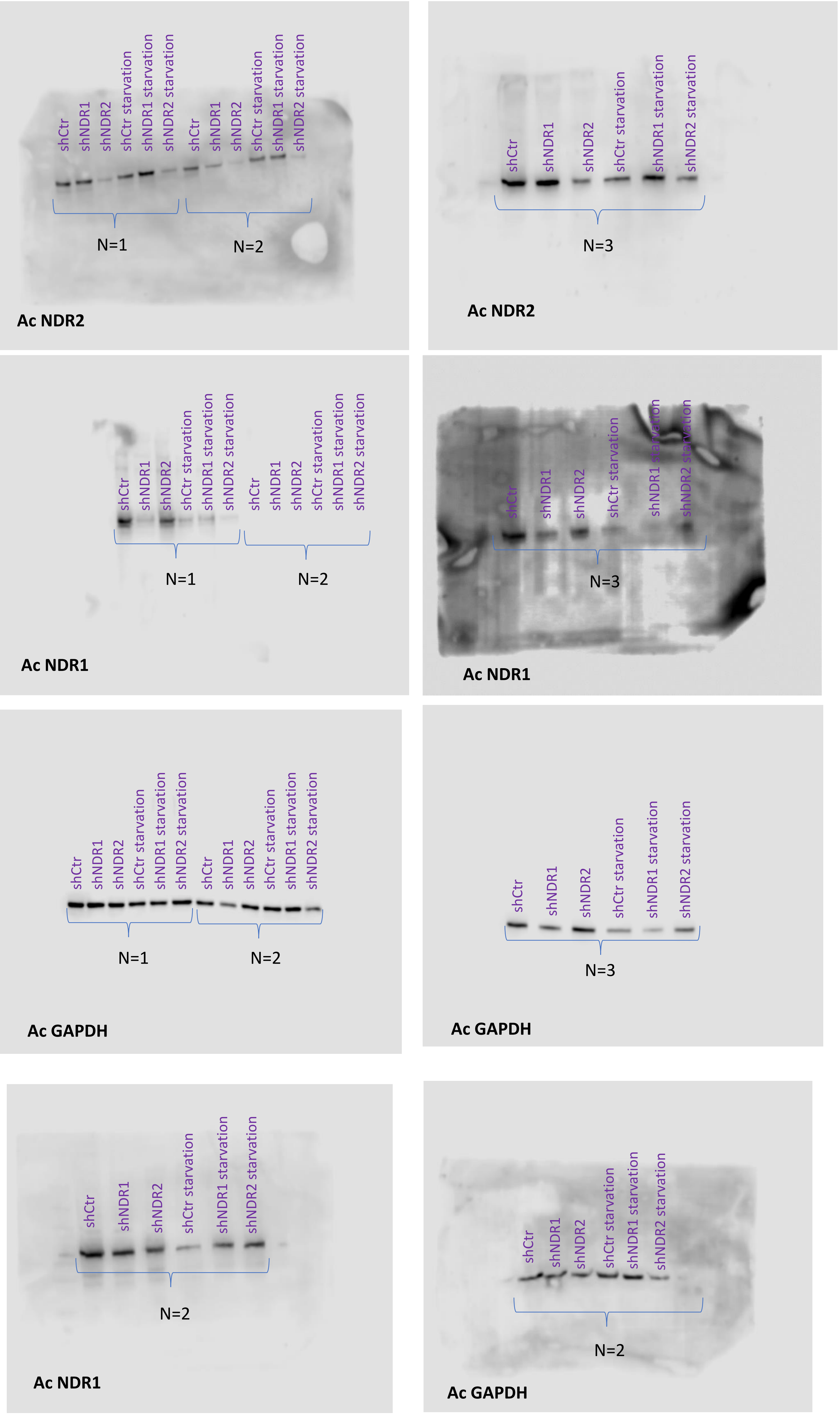

Figure 2G/H

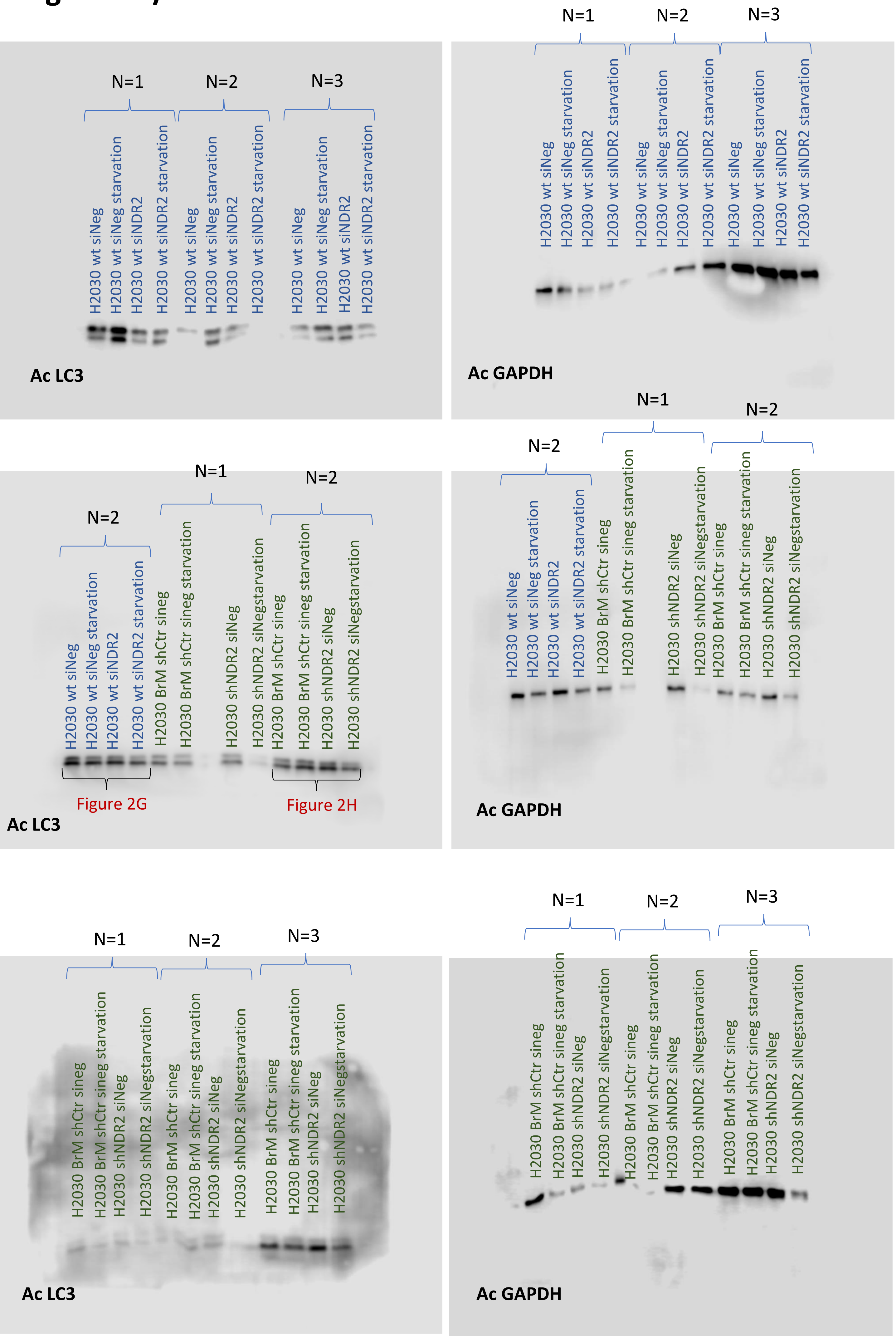
N=1
N=2
N=3
H2030 wt siNeg
H2030 wt siNeg starvation
H2030 wt siNDR2
H2030 wt siNDR2 starvation
H2030 wt siNeg
H2030 wt siNeg starvation
H2030 wt siNDR2
H2030 wt siNDR2 starvation
H2030 wt siNeg
H2030 wt siNeg starvation
H2030 wt siNDR2
H2030 wt siNDR2 starvation
Ac LC3
N=1
N=2
N=3
H2030 wt siNeg
H2030 wt siNeg starvation
H2030 wt siNDR2
H2030 wt siNDR2 starvation
H2030 wt siNeg
H2030 wt siNeg starvation
H2030 wt siNDR2
H2030 wt siNDR2 starvation
H2030 wt siNeg
H2030 wt siNeg starvation
H2030 wt siNDR2
H2030 wt siNDR2 starvation
Ac GAPDH
N=2
N=1
N=2
H2030 wt siNeg
H2030 wt siNeg starvation
H2030 wt siNDR2
H2030 wt siNDR2 starvation
H2030 BrM shCtr sineg
H2030 BrM shCtr sineg starvation
H2030 shNDR2 siNeg
H2030 shNDR2 siNegstarvation
H2030 BrM shCtr sineg
H2030 BrM shCtr sineg starvation
H2030 shNDR2 siNeg
H2030 shNDR2 siNegstarvation
Figure 2G
Figure 2H
Ac LC3
N=2
N=1
N=2
H2030 wt siNeg
H2030 wt siNeg starvation
H2030 wt siNDR2
H2030 wt siNDR2 starvation
H2030 BrM shCtr sineg
H2030 BrM shCtr sineg starvation
H2030 shNDR2 siNeg
H2030 shNDR2 siNegstarvation
H2030 BrM shCtr sineg
H2030 BrM shCtr sineg starvation
H2030 shNDR2 siNeg
H2030 shNDR2 siNegstarvation
Ac GAPDH
N=1
N=2
N=3
H2030 BrM shCtr sineg
H2030 BrM shCtr sineg starvation
H2030 shNDR2 siNeg
H2030 shNDR2 siNegstarvation
H2030 BrM shCtr sineg
H2030 BrM shCtr sineg starvation
H2030 shNDR2 siNeg
H2030 shNDR2 siNegstarvation
H2030 BrM shCtr sineg
H2030 BrM shCtr sineg starvation
H2030 shNDR2 siNeg
H2030 shNDR2 siNegstarvation
Ac LC3
N=1
N=2
N=3
H2030 BrM shCtr sineg
H2030 BrM shCtr sineg starvation
H2030 shNDR2 siNeg
H2030 shNDR2 siNegstarvation
H2030 BrM shCtr sineg
H2030 BrM shCtr sineg starvation
H2030 shNDR2 siNeg
H2030 shNDR2 siNegstarvation
H2030 BrM shCtr sineg
H2030 BrM shCtr sineg starvation
H2030 shNDR2 siNeg
H2030 shNDR2 siNegstarvation
Ac GAPDH

Figure 2G

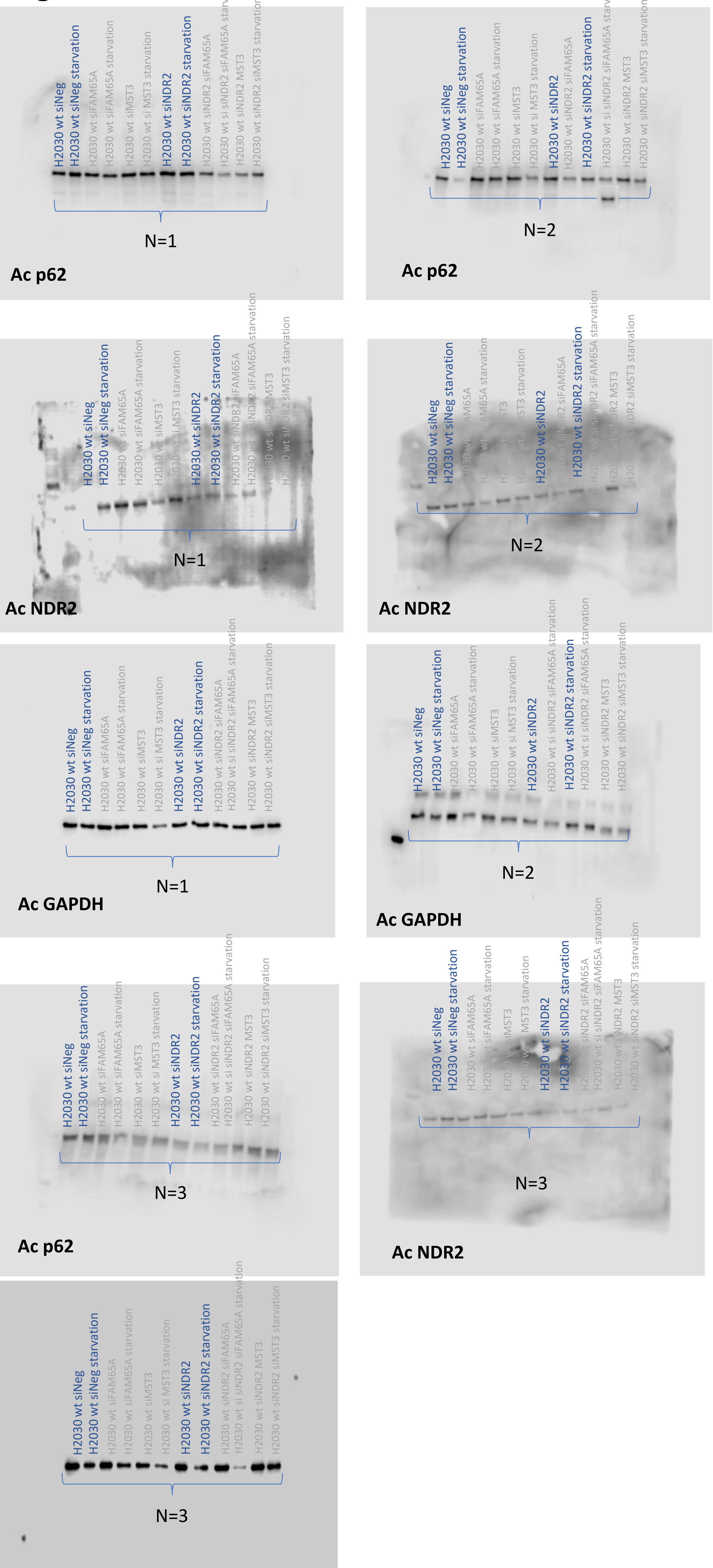
H2030 wt siNeg
H2030 wt siNeg starvation
H2030 wt siFAM65A
H2030 wt siFAM65A starvation
H2030 wt siMST3
H2030 wt si MST3 starvation
H2030 wt siNDR2
H2030 wt siNDR2 starvation
H2030 wt siNDR2 siFAM65A
H2030 wt si siNDR2 siFAM65A starvation
H2030 wt siNDR2 MST3
H2030 wt siNDR2 siMST3 starvation
N=1
Ac p62
N=2
Ac p62
N=1
Ac NDR2
N=2
Ac NDR2
N=1
Ac GAPDH
N=2
Ac GAPDH
N=3
Ac p62
N=3
Ac NDR2
N=3

Figure 2H

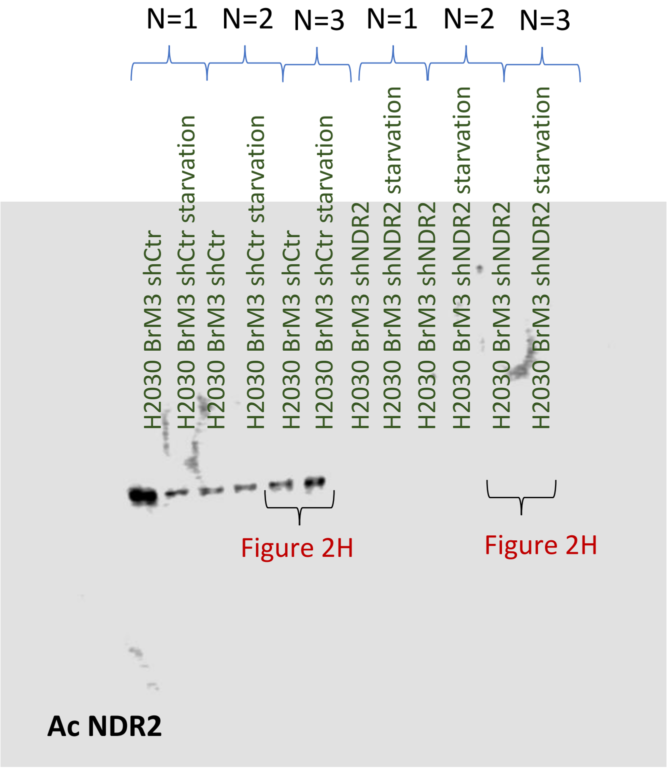
N=1 N=2 N=3 N=1 N=2 N=3
H2030 BrM3 shCtr
H2030 BrM3 shCtr starvation
H2030 BrM3 shCtr
H2030 BrM3 shCtr starvation
H2030 BrM3 shCtr
H2030 BrM3 shCtr starvation
H2030 BrM3 shNDR2
H2030 BrM3 shNDR2 starvation
H2030 BrM3 shNDR2
H2030 BrM3 shNDR2 starvation
H2030 BrM3 shNDR2
H2030 BrM3 shNDR2 starvation
Figure 2H
Figure 2H
Ac NDR2

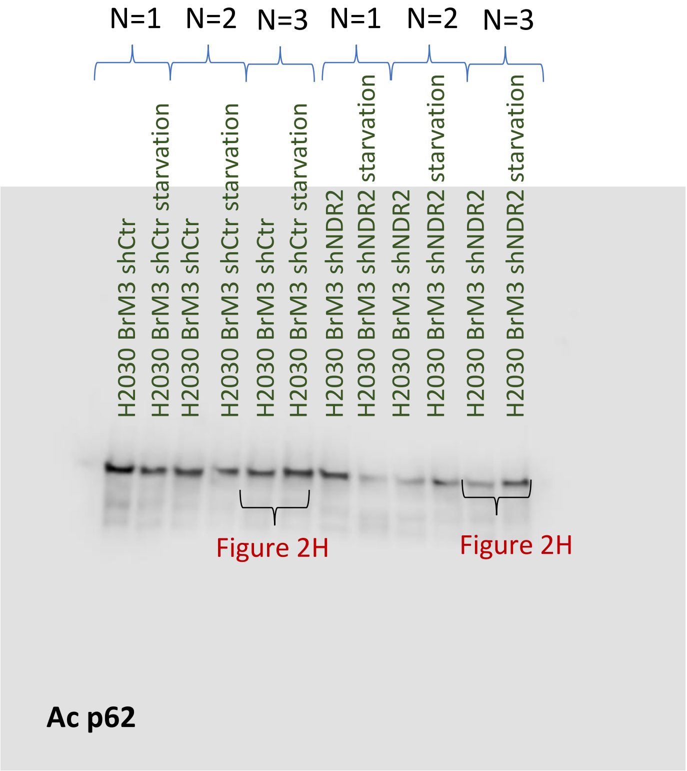
N=1 N=2 N=3 N=1 N=2 N=3
H2030 BrM3 shCtr
H2030 BrM3 shCtr starvation
H2030 BrM3 shCtr
H2030 BrM3 shCtr starvation
H2030 BrM3 shCtr
H2030 BrM3 shCtr starvation
H2030 BrM3 shNDR2
H2030 BrM3 shNDR2 starvation
H2030 BrM3 shNDR2
H2030 BrM3 shNDR2 starvation
H2030 BrM3 shNDR2
H2030 BrM3 shNDR2 starvation
Figure 2H
Figure 2H
Ac p62

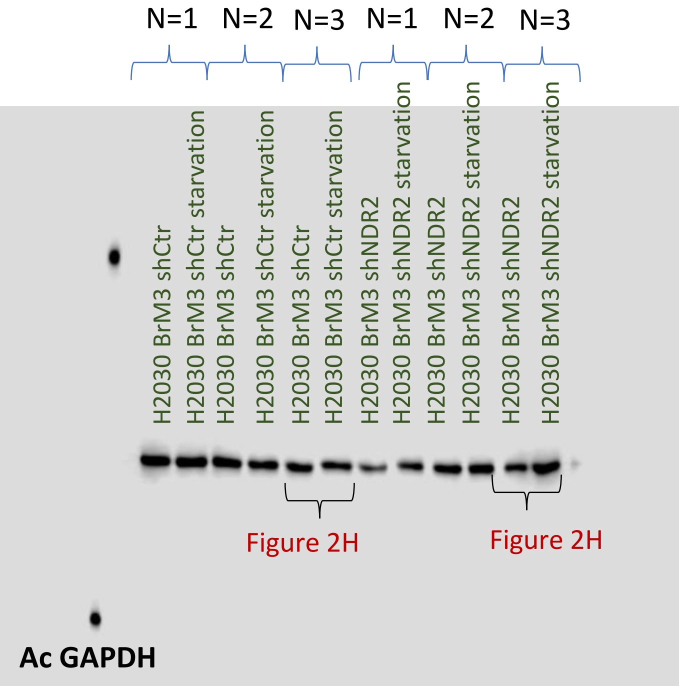
N=1 N=2 N=3 N=1 N=2 N=3
H2030 BrM3 shCtr
H2030 BrM3 shCtr starvation
H2030 BrM3 shCtr
H2030 BrM3 shCtr starvation
H2030 BrM3 shCtr
H2030 BrM3 shCtr starvation
H2030 BrM3 shNDR2
H2030 BrM3 shNDR2 starvation
H2030 BrM3 shNDR2
H2030 BrM3 shNDR2 starvation
H2030 BrM3 shNDR2
H2030 BrM3 shNDR2 starvation
Figure 2H
Figure 2H
Ac GAPDH

Figure 4F

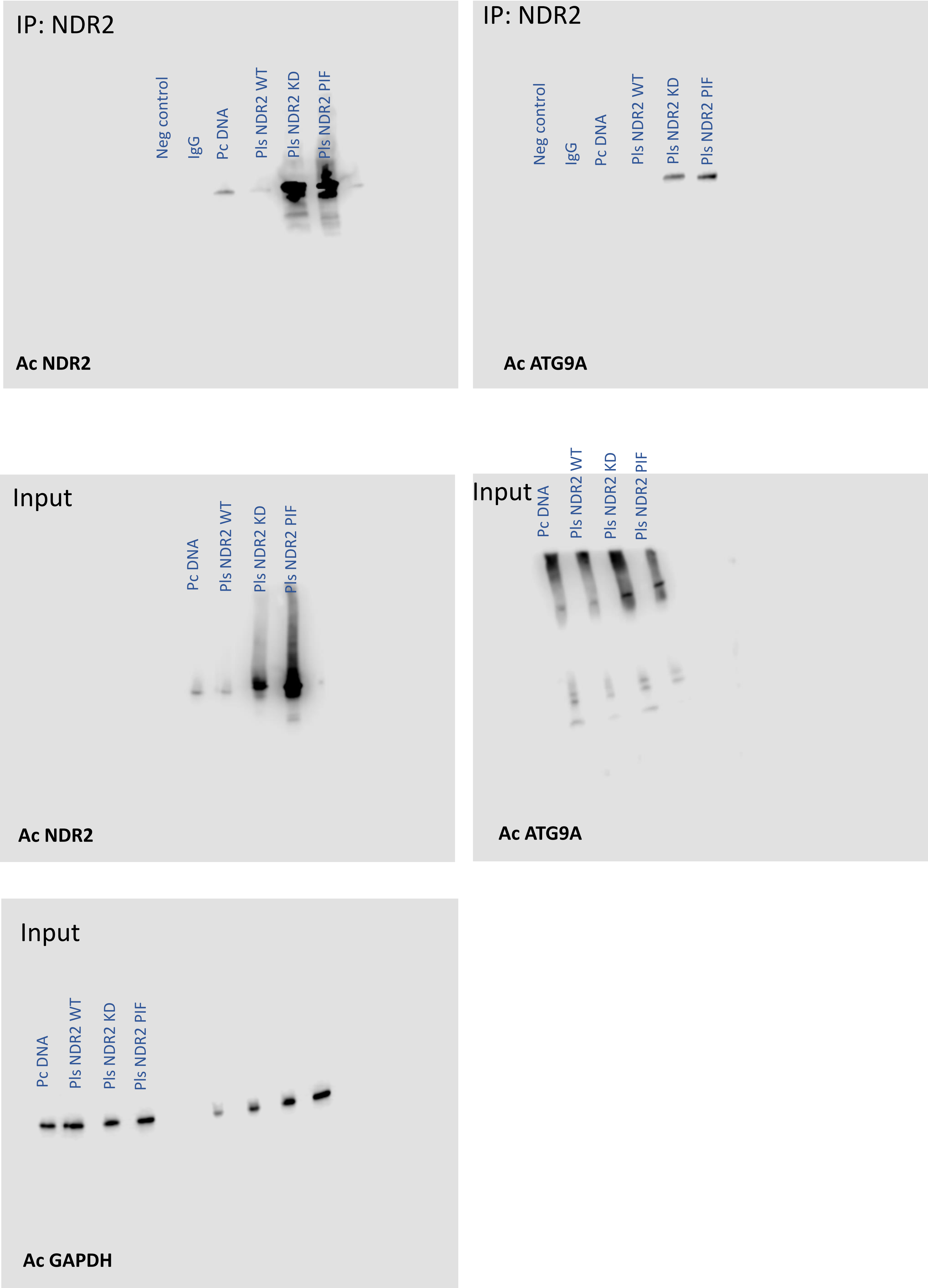
IP: NDR2
Neg control
IgG
Pc DNA
Pls NDR2 WT
Pls NDR2 KD
Pls NDR2 PIF
Ac NDR2
IP: NDR2
Neg control
IgG
Pc DNA
Pls NDR2 WT
Pls NDR2 KD
Pls NDR2 PIF
Ac ATG9A
Input
Pc DNA
Pls NDR2 WT
Pls NDR2 KD
Pls NDR2 PIF
Ac NDR2
Input
Pc DNA
Pls NDR2 WT
Pls NDR2 KD
Pls NDR2 PIF
Ac ATG9A
Input
Pc DNA
Pls NDR2 WT
Pls NDR2 KD
Pls NDR2 PIF
Ac GAPDH

Figure 4I

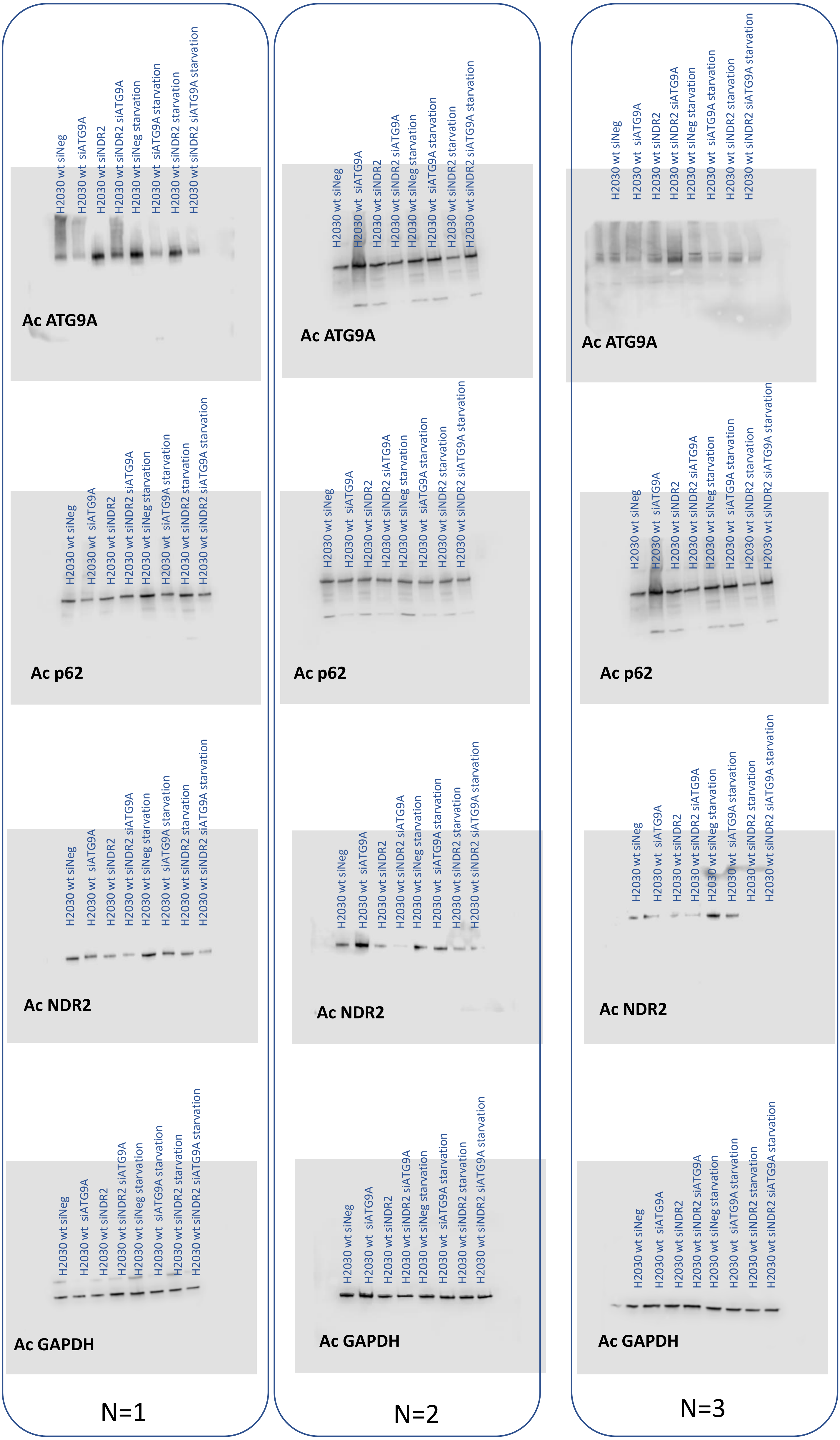
H2030 wt siNeg
H2030 wt siATG9A
H2030 wt siNDR2
H2030 wt siNDR2 siATG9A
H2030 wt siNeg starvation
H2030 wt siATG9A starvation
H2030 wt siNDR2 starvation
H2030 wt siNDR2 siATG9A starvation
Ac ATG9A
Ac p62
Ac NDR2
Ac GAPDH
N=1
N=2
N=3

Figure S2A

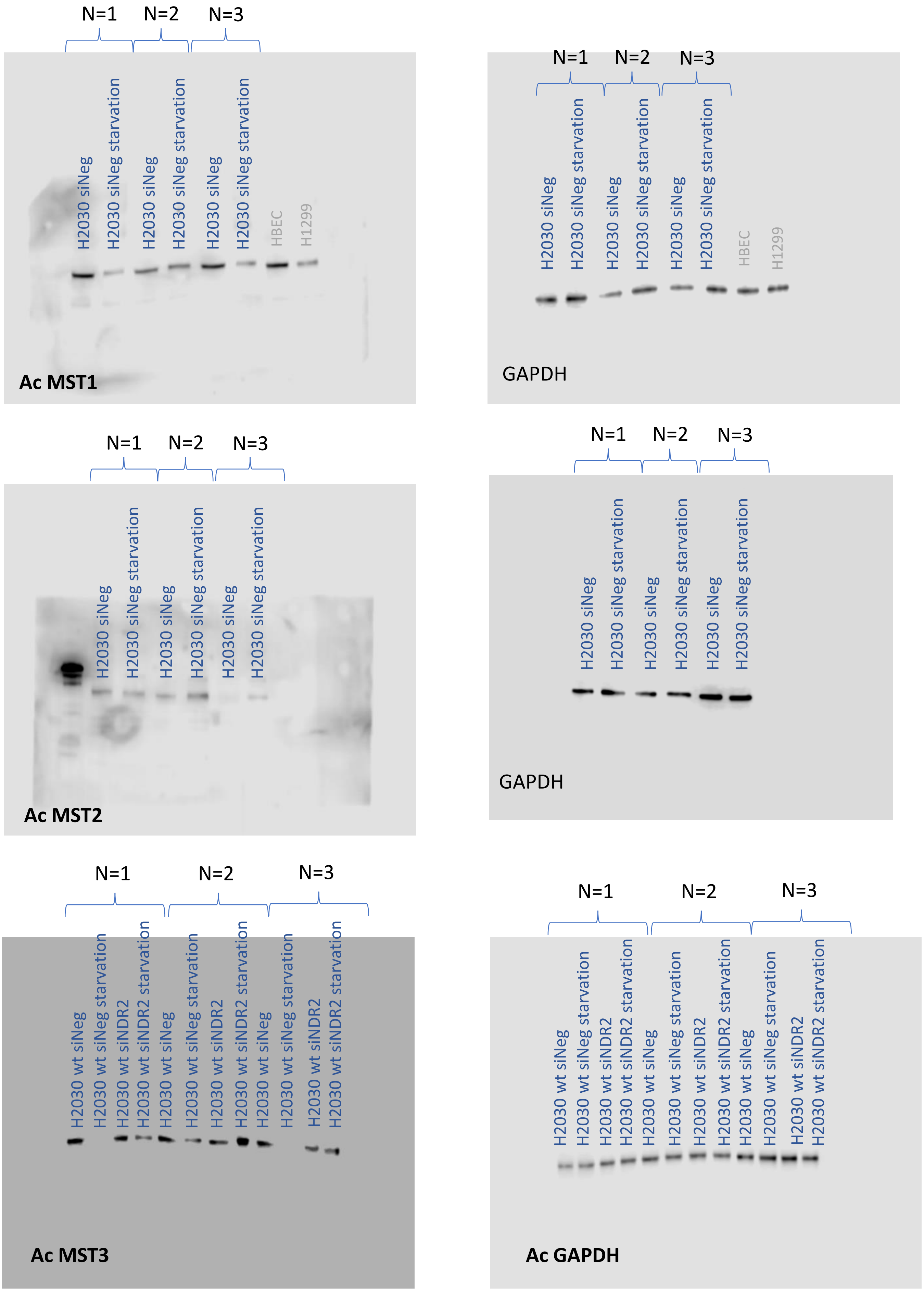
N=1
N=2
N=3
H2030 siNeg
H2030 siNeg starvation
H2030 siNeg
H2030 siNeg starvation
H2030 siNeg
H2030 siNeg starvation
HBEC
H1299
Ac MST1
N=1
N=2
N=3
H2030 siNeg
H2030 siNeg starvation
H2030 siNeg
H2030 siNeg starvation
H2030 siNeg
H2030 siNeg starvation
HBEC
H1299
GAPDH
N=1
N=2
N=3
H2030 siNeg
H2030 siNeg starvation
H2030 siNeg
H2030 siNeg starvation
H2030 siNeg
H2030 siNeg starvation
Ac MST2
N=1
N=2
N=3
H2030 siNeg
H2030 siNeg starvation
H2030 siNeg
H2030 siNeg starvation
H2030 siNeg
H2030 siNeg starvation
GAPDH
N=1
N=2
N=3
H2030 wt siNeg
H2030 wt siNeg starvation
H2030 wt siNDR2
H2030 wt siNDR2 starvation
H2030 wt siNeg
H2030 wt siNeg starvation
H2030 wt siNDR2
H2030 wt siNDR2 starvation
H2030 wt siNeg
H2030 wt siNeg starvation
H2030 wt siNDR2
H2030 wt siNDR2 starvation
Ac MST3
N=1
N=2
N=3
H2030 wt siNeg
H2030 wt siNeg starvation
H2030 wt siNDR2
H2030 wt siNDR2 starvation
H2030 wt siNeg
H2030 wt siNeg starvation
H2030 wt siNDR2
H2030 wt siNDR2 starvation
H2030 wt siNeg
H2030 wt siNeg starvation
H2030 wt siNDR2
H2030 wt siNDR2 starvation
Ac GAPDH
